# Actinomycetes in the spotlight: biodiversity and their role in bioremediation

**DOI:** 10.1007/s11274-025-04610-5

**Published:** 2026-01-17

**Authors:** Hagar S. Hashim, Mazen M. Zayan, Ahlam A. Mohamed, Hend Ismail Abd El rahman Abulila

**Affiliations:** 1 Faculty of Science, Microbiology Department, Helwan University, Cairo, 11795 Egypt; 2 Faculty of Agriculture, Cairo University, Program of Biotechnology, Giza, 12613 Egypt; 3 Faculty of Science, Chemistry and Microbiology Department, Cairo University, Giza, 12613 Egypt; 4 Faculty of Science (Girls Branch), Al-Azhar University, Cairo, Egypt

**Keywords:** Streptomycetes diversity, Actinomycetes taxonomy, Secondary metabolites, Detoxification, Microbial remediation

## Abstract

Actinomycetes represent a taxonomically and functionally diverse group of filamentous bacteria that are increasingly recognized for their role in environmental cleanup. Beyond their well-established capacity to produce antibiotics and other bioactive compounds, these microorganisms have shown remarkable potential in the degradation and transformation of a broad spectrum of pollutants, including petroleum hydrocarbons (e.g., crude oil, diesel), synthetic dyes (e.g., azo dyes), heavy metals (e.g., cadmium, lead), and pesticides (e.g., organochlorines). In recent years, significant progress has been made in uncovering novel actinomycete strains isolated from extreme and underexplored environments such as saline habitats, contaminated industrial sites, and marine sediments. These strains have demonstrated enhanced enzymatic activity and metabolic versatility in pollutant breakdown. Advances in molecular biology and omics-based techniques have further expanded our understanding of their biodegradative pathways and stress adaptation mechanisms. Nevertheless, several obstacles hinder their practical application, including inconsistent performance under field conditions, poor survival in competitive environments, and limited insight into their genetic regulation under contamination stress. This review not only summarizes current achievements (mainly from the last decade) but also highlights the urgent need for innovative strategies such as microbial consortia, metabolic engineering, and advanced formulation techniques to bridge the gap between laboratory findings and field-scale applications. Actinomycetes, therefore, stand as an untapped yet promising solution in the quest for efficient and eco-friendly bioremediation tools.

**Graphical abstract:**

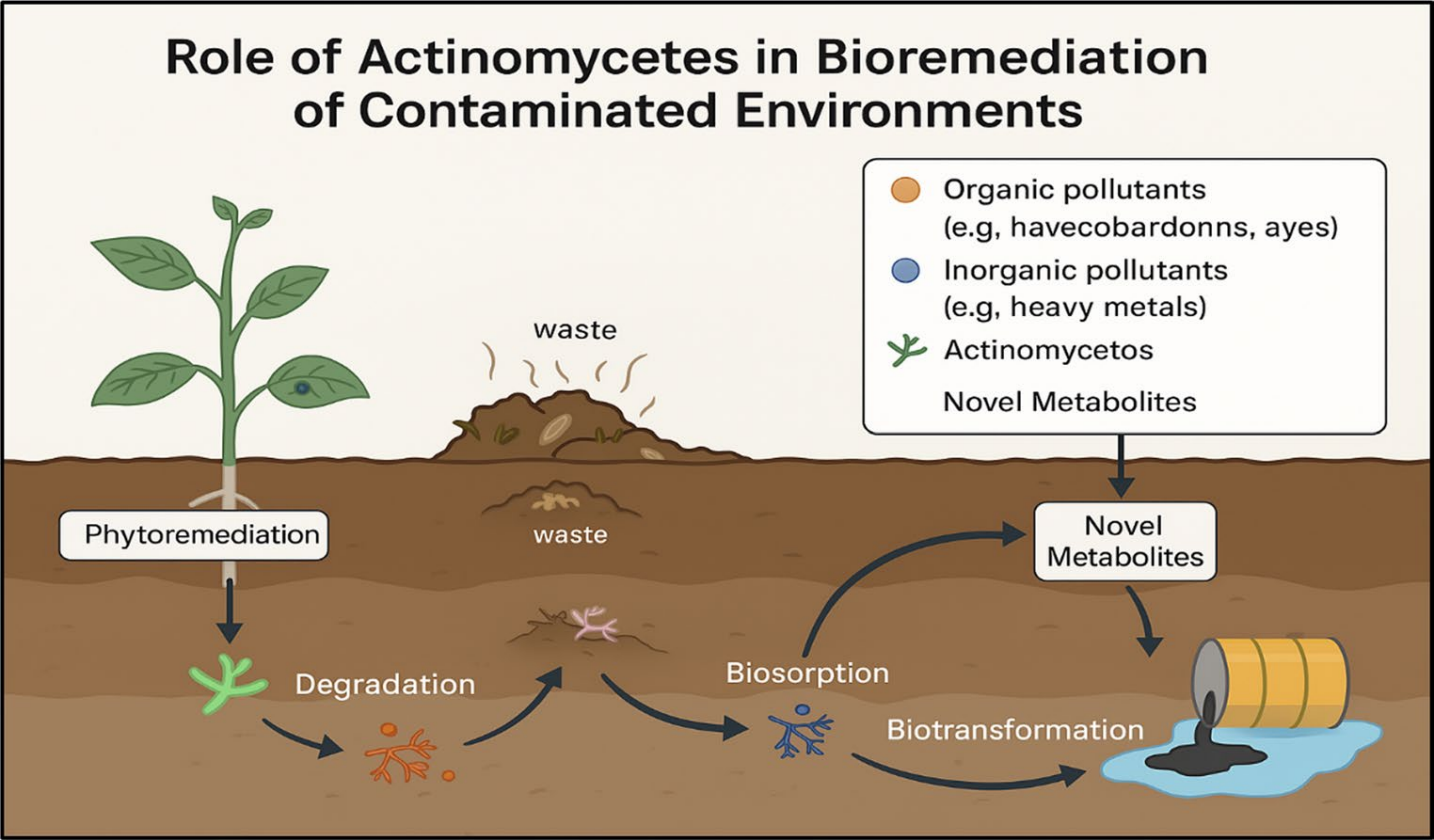

## Introduction

Historically, research on actinomycetes has primarily focused on their prolific capacity to produce antibiotics and other secondary metabolites, a field that gained momentum in the mid-20th century. However, by the 1980 s and 1990 s, their broader ecological significance began to unfold, with emerging studies revealing their ability to degrade complex organic pollutants such as lignin, polycyclic aromatic hydrocarbons (PAHs), and various xenobiotics. These early findings marked the beginning of a shift toward recognizing actinomycetes as potent agents of environmental bioremediation, setting the stage for current research efforts that delve deeper into their metabolic versatility, genetic potential, and adaptability to extreme conditions. While *Streptomyces* species have received significant attention for their role in biodegradation, the broader diversity of Actinobacteria including marine-derived and extremophilic genera remains underrepresented in current literature. Despite the increasing interest in the role of Actinobacteria in bioremediation, a comprehensive and up-to-date synthesis of their diversity and functional capabilities across different environments remains limited. Actinobacteria are primarily free-living bacteria that are extensively found in various natural habitats, including both aquatic and terrestrial ecosystems. These microorganisms exhibit remarkable survival strategies, allowing them to thrive under extreme environmental conditions high salinity, extreme pH, high temperature, and low nutrients. **(**Macagnan et al. 2006). Given their biotechnological significance such as production of industrial enzymes, secondary metabolites, biosurfactants, biocontrol agents, extensive research over the past five decades has focused on isolating novel Actinobacteria from various sources for drug discovery and screening programs **(**Selim et al. 2021).

The term “actinomycetes” is derived from the Greek words “aktis” (ray) and “mykes” (fungus), which highlight their filamentous structure, which shares similarities with both bacteria and fungi while maintaining distinct bacterial characteristics **(**Bhatti et al. 2017). These microorganisms are aerobic, spore-forming, Gram-positive bacteria characterized by a high guanine-cytosine (G + C) content ranging from 57% to 75% in their genomes. They belong to the order Streptomycetales and are characterized by the development of both substrate and aerial mycelia. Their filamentous growth resembles that of fungi, forming true aerial hyphae **(**Bhatti et al. 2017).

Actinobacteria, are renowned for their ability to produce a vast array of bioactive compounds, particularly antibiotics. Additionally, they are being explored for their potential applications in promoting plant growth and providing biocontrol in agriculture via consortia-based formulations, biofertilizers, or endophytic inoculants **(**Chaurasia et al. 2018).

While the majority of Actinobacteria are strictly aerobic, some taxa include facultative anaerobes that tolerate microaerophilic conditions. They are primarily chemoheterotrophic, relying on organic compounds as carbon and energy sources, and are particularly noted for their ability to degrade complex polysaccharides. **(**Barka et al. 2016).

Most Actinobacteria follow complex life cycles and are primarily saprophytic, thriving in soil environments. Historically, they were considered exclusively terrestrial; however, subsequent studies revealed their presence in aquatic habitats, initially attributed to the passive transport of terrestrial spores into marine environments (Prudence et al., 2020; Jagannathan et al. 2021). Today, Actinobacteria are recognized as highly versatile microorganisms, inhabiting diverse ecological niches, including soil and aquatic environments (e.g., *Streptomyces*,* Micromonospora*,* Rhodococcus*,* and Salinispora*), forming symbiotic relationships with plants (e.g., *Frankia* spp.), acting as pathogens in plants and animals (e.g., *Corynebacterium*, *Mycobacterium*, *Nocardia*), or residing as commensals in the gastrointestinal tract (e.g., *Bifidobacterium* spp.) **(**Barka et al. 2016).

Actinobacteria are abundant sources of new antimicrobial compounds such as, actinomycin D, rifamycins, and novel lantibiotics discovered in recent metagenomic studies **(**Atta et al. 2010). Many bioactive compounds derived from these bacteria have been identified through environmental screening programs that target diverse ecosystems, including soil and marine habitats **(**Duraipandiyan et al. 2010; Gallagher et al. 2010); Zotchev 2012**)**. Extensive research has led to the discovery and characterization of numerous novel actinomycete species, with secondary metabolites extracted using various isolation and screening techniques (Kim et al. 2011; Zotchev 2011).

*Streptomyces* strains were shown to degrade polycyclic aromatic hydrocarbons (PAHs) efficiently in a number of investigations; however, other findings indicated that their activity was restricted in salty settings. Targeted screening in saline-impacted regions may be necessary, since this disparity may be explained by variations in the source of isolation and the strains’ resistance to osmotic stress. Organic substances that are not directly involved in an organism’s main growth, development, or reproduction are known as secondary metabolites. Since Actinobacteria produce almost half of the known bioactive secondary metabolites, such as industrial enzymes, antibiotics, anticancer drugs, and anti-inflammatory chemicals, their metabolic variety highlights their significance in biotechnology.**(**Selim et al. 2021).

Beyond their pharmaceutical potential, Actinobacteria have been explored for producing nutraceuticals, enzyme inhibitors, and other industrially significant compounds **(**Zhao et al. 2016); Suriya et al. 2016). The enzymatic capabilities of Actinobacteria have been extensively studied, particularly for applications in waste management and environmental sustainability. For example, enzymes such as laccases, tyrosinases, and lignin peroxidases are employed in treating textile dyes and industrial wastewater. Additionally, amidases and esterases derived from *Nocardia* spp. They have been employed to improve the hydrophilicity of polyamide fibers and polyethylene terephthalate, providing environmentally friendly alternatives for the textile industry **(**Bettache et al. 2018). The ability of Actinobacteria to resist heavy metals, coupled with their metabolic versatility and distinctive growth traits such as filamentous mycelial development and rapid colonization of specific substrates highlights their potential as effective agents for bioremediation **(**Kannabiran 2017**).**

Actinobacteria are essential in biotechnology and pharmacology due to their exceptional ability to produce bioactive compounds, serving as a major source of antibiotics, antitumor agents, immunosuppressive drugs, and other therapeutics. **(**Chananan et al. 2023)

Although a number of reviews have already explored the bioremediation potential of Actinobacteria especially members of the genus *Streptomyces* (e.g., Romano et al. 2020); Behera and Das 2023; Rodríguez et al. 2021) many of these works have focused on narrow ecological scopes or specific applications such as salt-affected soils or polymer degradation. Moreover, several key reviews were not cited in recent syntheses, leading to redundant coverage and limited novelty. This review addresses these gaps by synthesizing diverse environmental sources, integrating recent insights from extremophilic and marine-derived genera, and proposing a framework that links ecological origin with functional bioremediation potential. This review offers an integrative exploration of their environmental distribution, functional capabilities, and underexploited potential in bioremediation across varied ecosystems.

While several reviews have addressed the biotechnological importance of Actinobacteria, most focus either on antibiotic production or general microbial diversity. This review distinguishes itself by providing an integrated overview of the diverse roles of Actinobacteria in the bioremediation of organic and inorganic pollutants, with emphasis on both foundational studies and more recent contributions. This review focuses on recent developments in bioactive metabolites obtained from terrestrial and marine Actinobacteria from 2016 to 2023, highlighting the methods used to discover and characterize new secondary metabolites. Unlike previous reviews, which predominantly focused on specific genera or isolated environments, the present work offers a broader comparative insight into Actinobacteria’ diversity, metabolic plasticity, and innovative perspectives, including genomic-driven bioprospecting.

## Biodiversity of actinobacteria

Actinobacteria are extensively found in diverse environments, acting as free-living saprophytes typically located in soil, water, and plants. They are an important source of biologically relevant bioactive compounds and are regularly evaluated for new bioactive materials. Significantly, Actinobacteria account for two-thirds of naturally occurring antibiotics **(**Yushchuk et al. 2020). Soil is considered the primary habitat for diverse actinomycete populations, with their composition varying depending on soil type. Literature reviews regarding the isolation of Actinobacteria indicate that only 10% of these microorganisms have been successfully extracted from natural environments for the purpose of antibiotic discovery **(**Cardenas-de la Garza et al. 2020). Leveraging foundational knowledge of their habitats has facilitated advancements in understanding Actinobacteria’ physiology, biomolecule production, and ecological significance, enabling exploration in unique environments. The Manipur region, located within the Indo-Myanmar biodiversity hotspot, is a promising reservoir of novel actinomycete species, including *Actinoplanes*, *Micromonospora*, *Actinomadura*, *Nonomuraea*, *Streptosporangium*, and *Nocardia* **(**Guo et al. 2015). Marine Actinobacteria are considered highly significant due to their role in biotechnological and biological applications, contributing to over 50% of all known bioactive secondary metabolites found in nature **(**Katarzyn et al. 2018).

Marine Actinobacteria represent a particularly rich source of novel secondary metabolites, although their contribution is not as quantitatively dominant as sometimes suggested. Overall, Actinobacteria are responsible for nearly 70% of all known microbial secondary metabolites (Bérdy 2023). Within this group, marine-associated genera such as *Streptomyces*, *Micromonospora*, and *Nocardiopsis* have yielded hundreds of structurally diverse compounds, underscoring their disproportionate importance for natural product discovery despite a lower numerical share compared to terrestrial strains (Manivasagan et al. 2023; Subramani and Aalbersberg 2024).

Marine Actinobacteria have been classified into specific genera based on their chemical and morphological traits, and they are vital for the degradation of organic matter. Actinobacteria encompass a range of genera such as *Corynebacterium*,* Actinomyces*,* Frankia*,* Arthrobacter*,* Micrococcus*,* Micromonospora*, and *Streptomyces*, with the latter being the most isolated and recognized as a prolific producer of biologically active secondary metabolites **(**Roshan et al. 2014). Despite their potential, marine Actinobacteria remain largely unexplored due to the challenges of isolating them from their natural oceanic habitats. Nevertheless, their cultivation methods remain consistent. These microorganisms thrive in unique marine environments such as deep-sea hydrate reservoirs and marine organic aggregates, where they demonstrate considerable antagonistic activity within microbial communities **(**Singh et al. 2014).

Extensive research has been conducted on pigmented Actinobacteria and their biological activities. Certain microbial pigments have been reported to possess anticancer, antiviral, anti-inflammatory, and antioxidant properties. These pigments exhibit stability under diverse conditions of heat, light, and pH, making them suitable for applications in food, cosmetics, textiles, and pharmaceuticals **(**Ibrahim et al. 2023). Freshwater habitats also represent a valuable reservoir of Actinobacteria, known for producing important bioactive secondary metabolites. *Streptomyces* predominate in river water, whereas *Micromonospora* is primarily found in river sediments. Actinobacteria isolated from freshwater environments exhibit notable antifungal activity **(**Kim et al. 2005).

Desert habitats are recognized as an underexplored source of novel antibacterial diversity, with numerous new bacterial species identified from samples collected in the Atacama Desert. Due to high oxidation levels, soil in these hyper-arid regions lacks organic material, resulting in a low cultivable bacterial population. Actinobacteria extracted from desert soil exhibit antimicrobial properties, indicating that desert environments could contain a substantial population of alkaliphilic and halophilic Actinobacteria **(**Jiang et al. 2015). Similarly, hypersaline habitats, such as salterns, saline lakes, hypersaline soils, and saline environments, represent extreme ecosystems **(**Khanna et al. 2011). Hypersaline soils generally have a salt content of 9–23%, and the actinomycete genera found in these environments include *Streptoverticillium*,* Streptomyces*,* Micromonospora*,* Microbispora*,* Nocardia*,* Actinoplanes*,* Kitasatosporia*,* Planomonospora*, as well as *Streptomyces albidoflavus*, *Streptomyces alboflavus*,* Streptomyces griseoflavus*,* and Streptomyces rimosus*. These Actinobacteria establish stable dominant populations in both the rhizosphere and bulk soil during the winter and spring seasons **(**Kumar et al. 2010; Duangmal et al. 2012). Actinobacteria play a crucial role in rhizospheres, enhancing plant growth and providing protection against phytopathogens.

Actinobacteria have evolved a range of adaptive strategies to endure thermal stress, such as the production of chaperones that aid in the refolding of partially denatured proteins, a high GC content, amino acid substitutions in proteins, specific cell wall components, and a higher ratio of charged amino acids (Asp, Glu, Arg, and Lys) compared to polar amino acids (Asn, Gln, Ser, and Thr). Numerous studies have reported diverse Actinobacterial populations in thermal environments, including analyses of culturable Actinobacteria from hot spring samples (40–99 °C) in Tengchong County, Yunnan Province, China. Among the 58 thermophilic Actinobacterial species identified were *Actinomadura*,* Microbispora*,* Micromonospora*,* Micrococcus*,* Nonomuraea*,* Nocardiopsis*,* Promicromonospora*,* Pseudonocardia*,* Streptomyces*,* Thermoactinospora*,* Thermocatellispora*, and *Verrucosispora. Streptomyces* was the most commonly isolated genus, although Actinobacterial diversity was greater at lower temperatures **(**Rao and Li 2022**).**

The potential of Actinobacteria in bioremediation is a growing research focus, especially given increasing concerns about industrial pollution in soil and water systems. There is a rising demand for effective methods to combat contamination from pesticides, heavy metals, and mixed pollutants. Existing techniques for removing these pollutants often lack efficacy, particularly for inorganic compounds. While organic pollutants can be fully degraded into harmless substances, inorganic pollutants often persist as toxic intermediates (Jagannathan et al. 2021). Actinobacteria are widely recognized as natural biocontrol agents in soil ecosystems. Actinobacteria, in particular, exhibit enzymatic capabilities that enhance soil productivity by promoting nutrient cycling, decomposing complex polysaccharides, restoring soil health, and serving as primary defenses against soil-borne pathogens. Additionally, Actinobacteria affect root architecture by restraining primary root elongation while encouraging lateral root development, which in turn improves nutrient uptake **(**Javed et al. 2020).

To better understand the ecological diversity and functional significance of Actinobacteria, it is essential to examine their distribution across diverse natural habitats and identify the most frequently associated genera and species. Table 1 presents representative examples of Actinobacteria isolated from a variety of environments such as soil, marine ecosystems, extreme habitats, and polluted sites along with the typical techniques used for their isolation. This comprehensive overview highlights the remarkable adaptive versatility of Actinobacteria and provides a useful framework for guiding targeted bioprospecting and ecological studies.

**Table 1:** Ecological distribution of actinomycetes across various habitats, highlighting representative genera, species, and commonly used isolation techniques

Habitat	Isolation techniques	Major genera	Representative species
Forest and agricultural soil	Starch casein agar, humic acid-vitamin media	*Streptomyces*, *Nocardia*	*S. griseus*, *S. coelicolor*
Marine sediment	Heat pre-treatment, seawater-based media	*Salinispora*, *Micromonospora*	*S. tropica*, *M. marina*
Compost and manure	Serial dilution, pH/temperature-selective techniques	*Thermoactinomyces*, *Actinomadura*	*T. vulgaris*, *A. madurae*
Rhizosphere	Root washing, CaCO₃-enriched media	*Streptomyces*, *Pseudonocardia*	*S. lydicus*, *P. autotrophica*
Extreme environments (desert, saline soil, hypersaline marine)	Dry heat, saline-based media	*Nocardiopsis*, *Actinopolyspora*	*N. dassonvillei*, *A. halophila*

Actinobacteria have been isolated from a wide range of ecosystems, including terrestrial (soil, rhizosphere), aquatic (marine and freshwater), and extreme environments (deserts, arid lands, hot springs, and polar regions). However, there is a noticeable geographic and ecological bias in the current literature, with most studies disproportionately focused on Actinobacteria from agricultural and forest soils in tropical and subtropical regions, particularly in Asia and Latin America **(**Barka et al. 2016; Genilloud 2017**).** This overrepresentation potentially overlooks unique and metabolically diverse strains that may inhabit underexplored niches such as deep-sea sediments **(**Subramani and Aalbersberg 2012**)**, polar soils **(**Zhang et al. 2019), or saline and alkaline ecosystems **(**Qin et al. 2011).

Moreover, traditional culture based methods, which are still predominantly used in biodiversity surveys, fail to recover a significant portion of the actinomycetal community particularly the rare and slow-growing taxa **(**Tiwari and Gupta 2012**).** Recent advances in high throughput sequencing and metagenomic approaches offer powerful alternatives for capturing uncultured diversity, yet these methods are still underutilized in actinomycete biogeographic studies **(**Goodfellow and Fiedler 2010; Tanaka et al. 2018).

There is an urgent need to expand sampling efforts to neglected ecosystems and adopt modern molecular tools to obtain a more accurate picture of actinomycete global diversity. Such approaches could facilitate the discovery of novel strains with unique metabolic traits, including those with bioremediation potential under extreme or contaminated conditions. Future studies should also focus on understanding the ecological drivers of actinomycete distribution, such as pH, salinity, and anthropogenic pollution, to better link biodiversity with functional capacities.

## Taxonomy

### Classification

The phylum Actinobacteria has undergone a reorganisation in categorisation, with the following six classes identified in Volume 5 of Bergey’s Manual of Systematic Bacteriology (2nd edition): Actinobacteria, *Acidimicrobiia*,* Coriobacteriia*,* Nitriliruptoria*,* Rubrobacteria*,* and Thermoleophilia*. These classes include internationally recognized names and descriptions of bacteria. The class Actinobacteria comprises 16 orders: *Actinomycetales*,* Actinopolysporales*,* Bifidobacteriales*,* Catenulisporales*,* Corynebacteriales*,* Frankiales*,* Glycomycetales*,* Jiangellales*,* Kineosporiales*,* Micrococcales*,* Micromonosporales*,* Propionibacteriales*,* Pseudonocardiales*,* Streptomycetales*,* and Streptosporangiales. Streptomyces*, *Nocardia*, and *Micromonospora* are the most common genera of Actinobacteria in soil habitats. But *Streptosporangium*, *Micromonospora*, and *Actinoplanes* are also commonly seen. **(**Devanshi et al. 2021). Actinobacteria is one of the largest taxonomic groups among the primary families that are currently recognised within the domain *Bacteria*. **(**Ludwig et al. 2012**).** To date, researchers have sequenced the genomes of Actinobacterial species that are significant to biotechnology, ecology, and both human and veterinary medicine. The observed genomic heterogeneity of these organisms is believed to reflect their biodiversity **(**Ventura et al. 2007). Actinobacteria continue to be one of the largest taxonomic groups. **(**Ludwig et al. 2012**).**

**Fig. 1:**
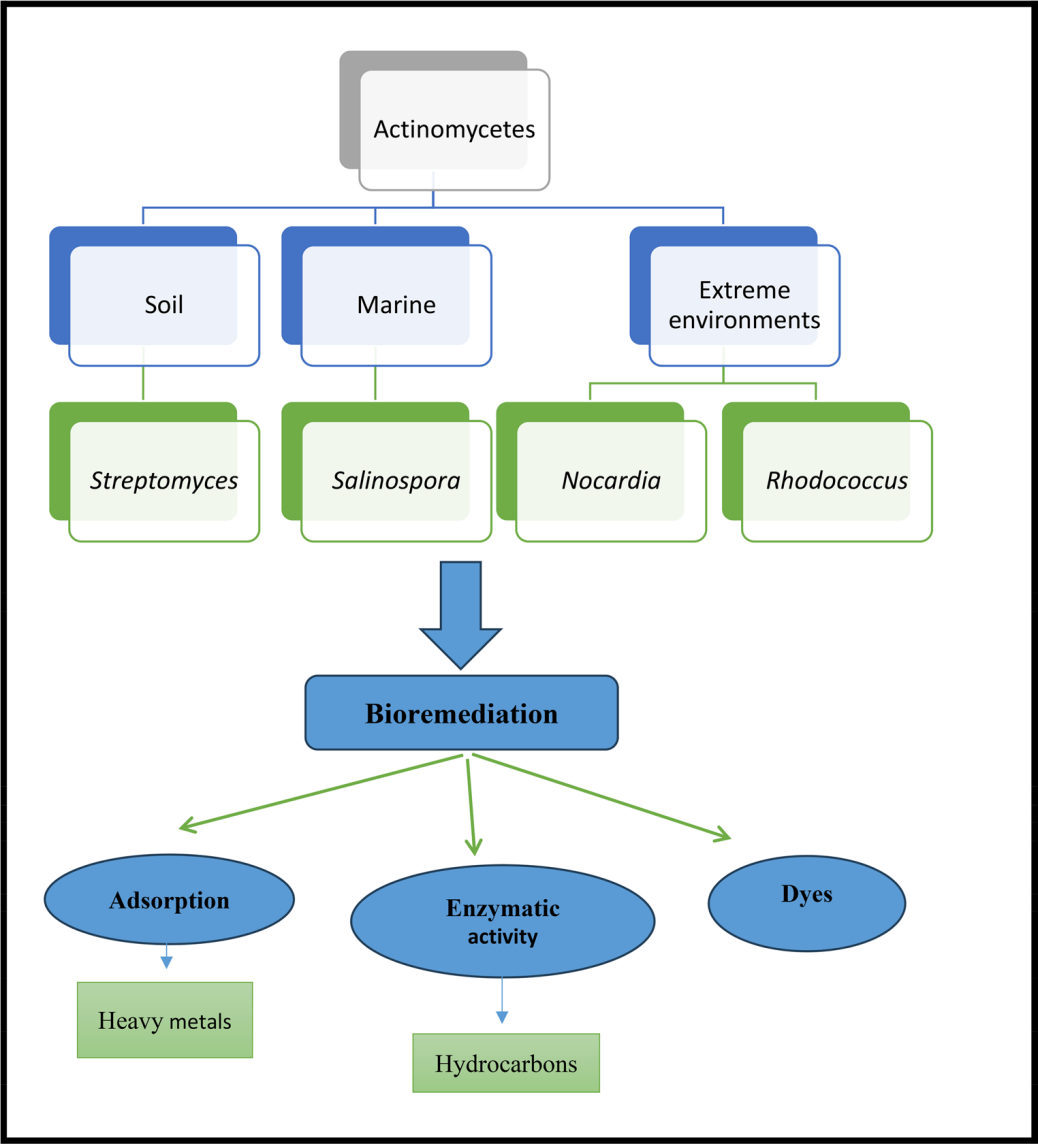
Taxonomic structure of the *Actinobacteria* phylum with its habitats and bioremediation

According to recent research, Actinobacteria belong to the phylum Actinobacteria, a major lineage of Gram-positive bacteria characterized by a high G + C content. Within this phylum, the class Actinobacteria includes several ecologically and industrially significant orders, such as *Actinomycetales*, *Streptomycetales*, *Micromonosporales*, and *Pseudonocardiales*. These orders encompass a broad diversity of genera known for their metabolic versatility and environmental relevance. Among them, the genus *Streptomyces* is the most extensively studied, particularly due to its remarkable capacity for producing antibiotics and its involvement in organic matter degradation. In addition, other genera such as *Nocardia*, *Micromonospora*, *Rhodococcus*, and *Nocardiopsis* have demonstrated notable capabilities in bioremediation, especially in degrading complex organic pollutants and resisting heavy metals, as illustrated in Fig. 1.

Table 2 presents the hierarchical taxonomic classification of Actinobacteria, highlighting their representative roles in processes such as biodegradation, bioremediation, and metal resistance. Notably, their high G + C genomic content and filamentous growth morphology are distinguishing features that contribute to their ecological adaptability.

**Table 2:** Taxonomic classification of actinomycetes and their roles in bioremediation

Taxonomic rank	Example name(s)	Notable role in bioremediation
Phylum	*Actinobacteria*	High G + C Gram-positive bacteria; major source of biodegradative strains
Class	*Actinobacteria*	Filamentous morphology; spore-forming; stress-resistant
Order	*Streptomycetales*, *Micromonosporales*, *Pseudonocardiales*, *Actinomycetales*	Production of extracellular enzymes and secondary metabolites; pollutant degradation
Family	*Streptomycetaceae*, *Nocardiaceae*, *Micromonosporaceae*, *Pseudonocardiaceae*	Environmental detoxification, metal resistance, hydrocarbon degradation
Genus	*Streptomyces*, *Rhodococcus*, *Nocardia*, *Micromonospora*, *Actinoplanes*, *Pseudonocardia*, *Nocardiopsis*, *Saccharopolyspora*	Degradation of hydrocarbons, pesticides, dyes, and heavy metals

The biodiversity of Actinobacteria is vast, spanning multiple genera, ecological niches, and metabolic profiles. While the genus *Streptomyces* remains the most extensively studied due to its prolific production of antibiotics and enzymes, recent research has uncovered a wealth of diversity among lesser-known genera such as *Micromonospora*, *Actinoplanes*, *Pseudonocardia*, and *Saccharopolyspora* **(**Baltz 2017; Nouioui et al. 2018). These rare or “non-streptomycete” Actinobacteria are increasingly recognized for their unique metabolic capabilities, including novel biosynthetic gene clusters not found in *Streptomyces*.

However, the current understanding of actinomycete biodiversity is still limited by methodological constraints. Most diversity assessments rely on 16 S rRNA gene sequencing, which, while useful for broad taxonomic classification, lacks the resolution to uncover functional diversity or closely related species **(**Goodfellow et al. 2012). Advances in whole-genome sequencing, metagenomics, and comparative genomics now offer more accurate tools to explore the true extent of actinomycete diversity, yet their application remains scarce, particularly in non-model genera or environmental samples **(**Choudoir et al. 2016).

Another overlooked aspect is the role of environmental and evolutionary pressures in shaping actinomycete diversity. Factors such as competition, horizontal gene transfer, and local adaptation to extreme or polluted environments likely contribute to the evolution of specialized strains with distinct biodegradation or antibiotic profiles. Integrating ecological theories and molecular tools could provide new perspectives on how Actinobacteria diversify and specialize **(**Aigle et al. 2014).

A critical gap lies in linking taxonomic diversity with functional outcomes. For example, while many studies isolate and identify diverse Actinobacteria from soils or marine sediments, few go further to characterize their metabolic pathways or biotechnological potential in situ. Future research should combine taxonomic surveys with functional profiling (e.g., metabolomics, transcriptomics) to better harness this microbial resource.

### Morphology

**Fig. 2:**
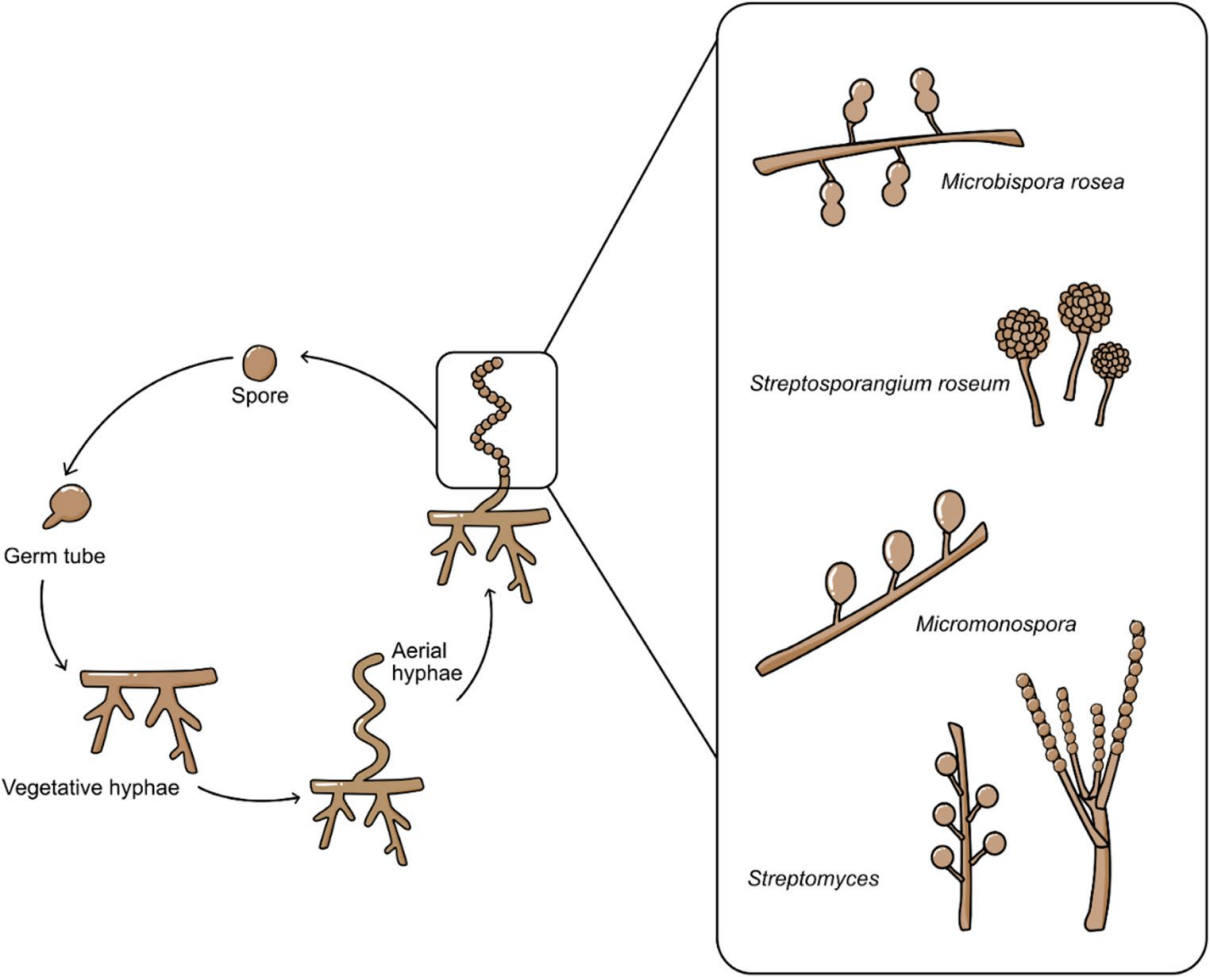
The stages of actinomycetes’ life cycle, from conidiospores to sporulation. The expanded box displays the numerous conidiospore types

The main characteristics used to classify Actinobacteria at the genus and species levels are microscopic morphology and chemotaxonomy. For a more precise classification, menaquinone type and phospholipid composition may also be taken into account, but the latter primarily focuses on the makeup of the cell wall and the distribution of sugars across the entire cell. (Labeda et al., 1987). Although the majority of Actinobacteria are aerobic, saprophytic microorganisms with intricate life cycles Fig. 2, there are always exceptions. (Prudence et al., 2020)

**Fig. 3:**
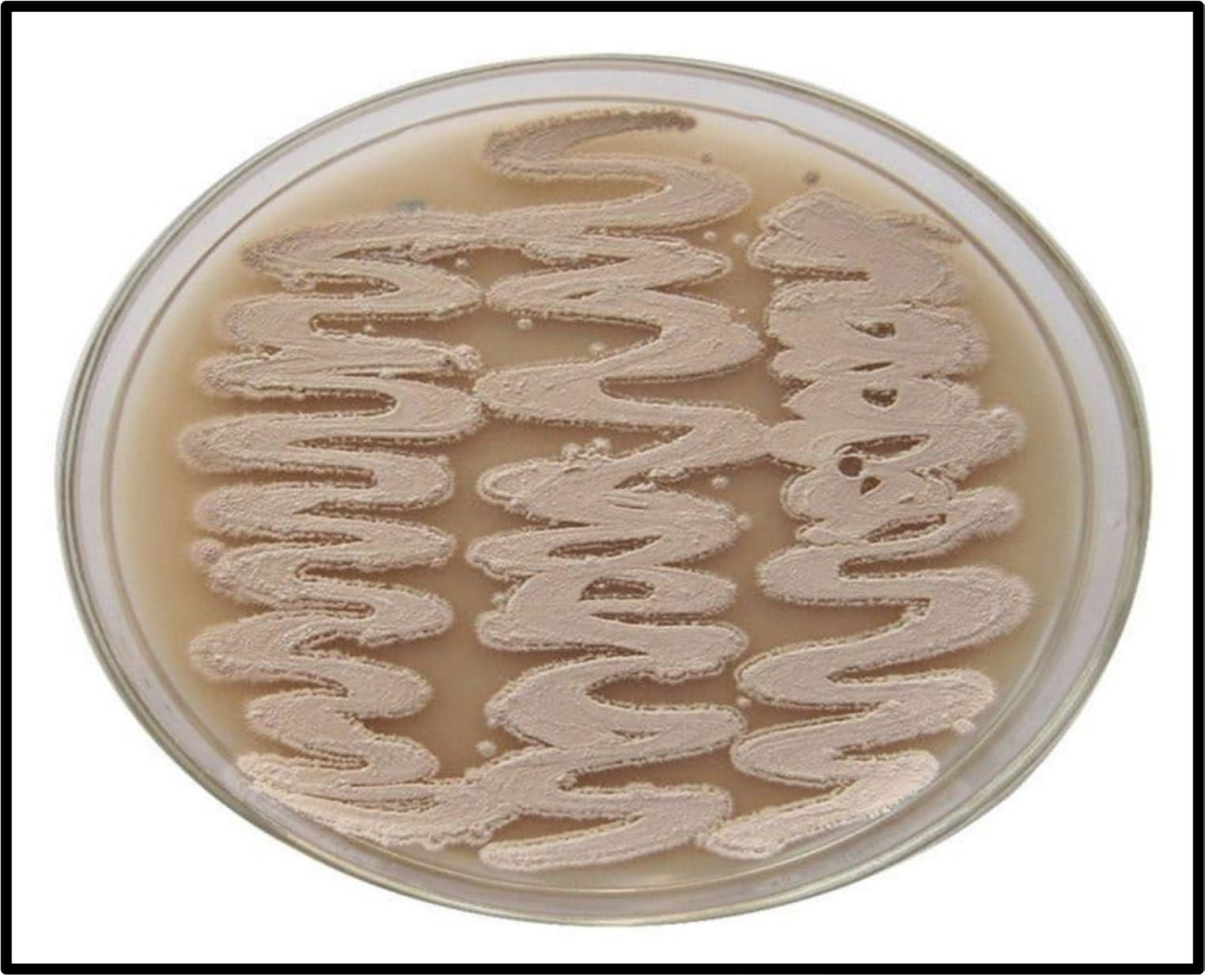
Aerial Streptomyces sp. mycelium colour. (El-Naggar et al. 2016)

**Fig. 4:**
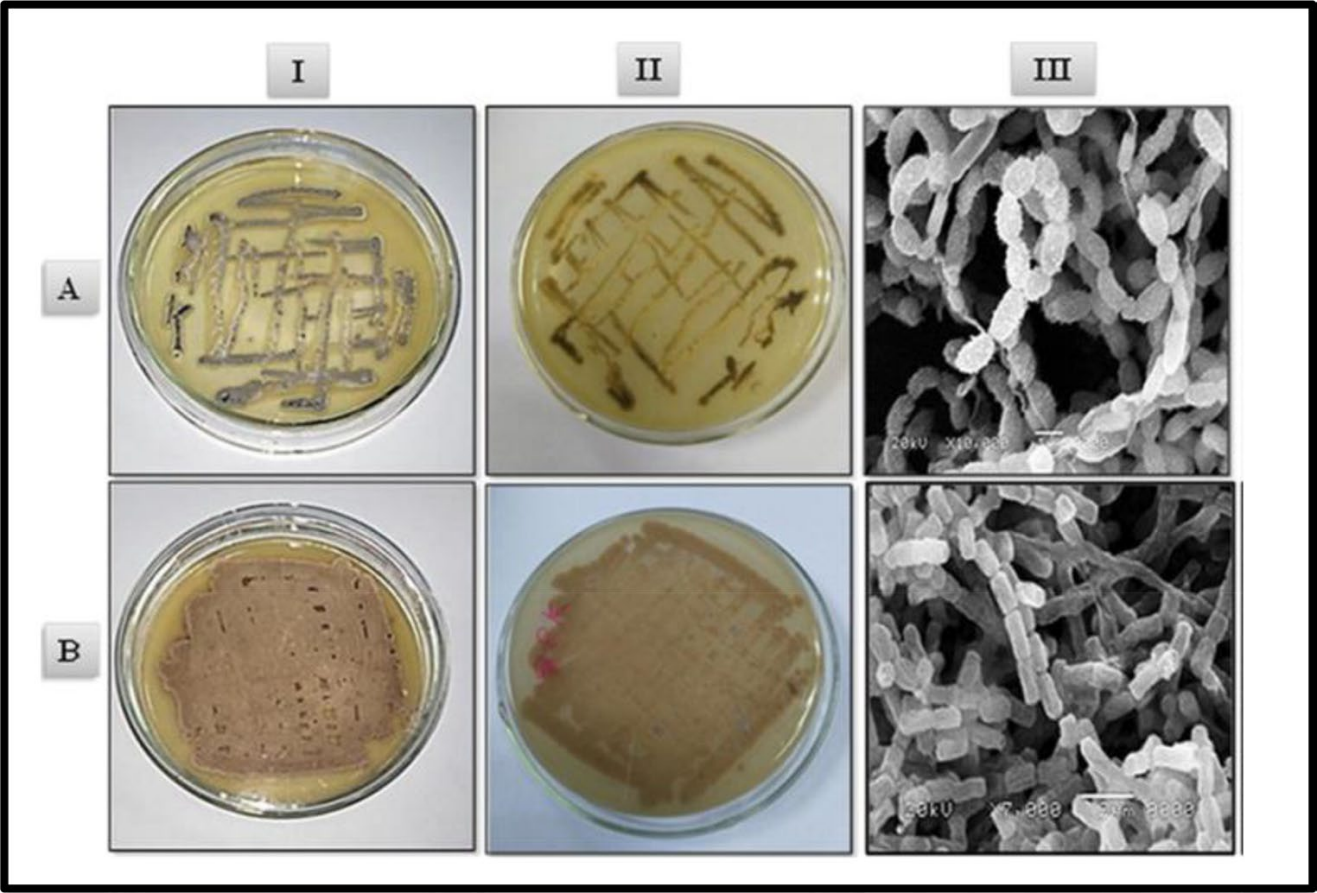
Isolate Streptomyces variabilis 4NC (I) aerial mycelium (II) substrate mycelium(III) scanning electron micrograph showing spiral spore chain with spiny surface, b Isolate S. fradiae 8PK (I) aerial mycelium, (II) substrate mycelium, (III) scanning electron micrograph showing straight spore chain with smooth surface. (Tolba et al. 2019)

A specialized type of vegetative reproduction in Actinobacteria is mycelial fragmentation. However, Actinobacteria that primarily exhibit a mycelial lifestyle usually reproduce by producing asexual spores. The key morphological differences among Actinobacteria include their spores’ form and appearance, the development of diffusible melanoid pigments, mycelial colour, and the existence or lack of aerial mycelium or substrate.The color of the *Streptomyces* sp. aerial mycelium after 7–14 days of incubation at 30 °C on starch-nitrate agar medium can be observed in Fig. 3. Furthermore, Fig. 4 illustrates the differences in the spore chain formation, colour, and growth of Actinobacterial isolates on starch-nitrate agar media made with seawater.

### Phylogenetic characters

**Fig. 5:**
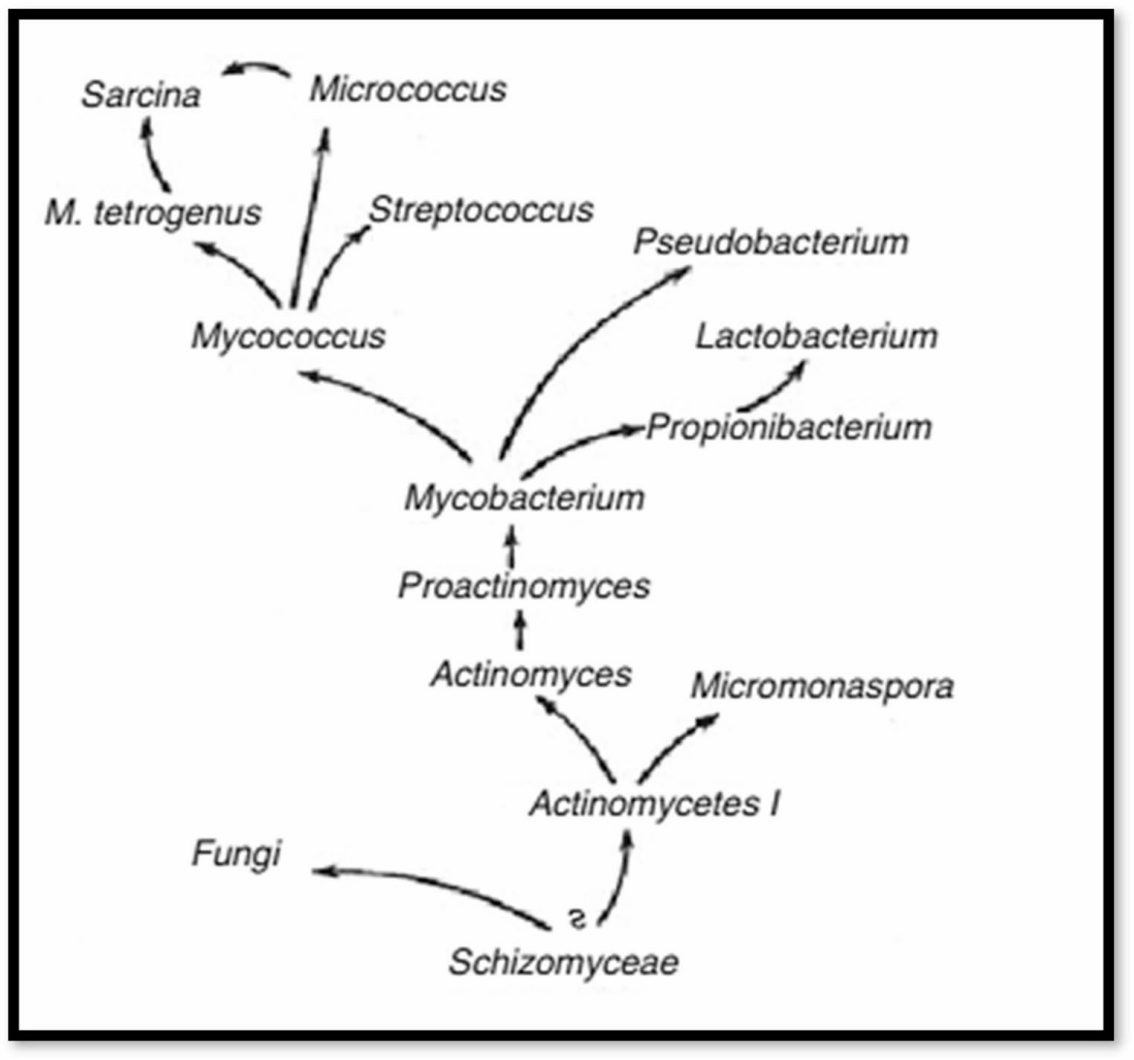
An illustration of the morphological and physiological characteristics that actinomycetes and related species have in common (Krassil’nikov  1959)

Alternative molecular markers used in Actinobacteria phylogeny are limited to definite groups within this phylum. Phylogenetic interactions between certain groups of Actinobacteria, such as *Streptomyces* members, mycobacteria, and bifidobacteria, have been constructed using the *RpoB*,* RecA*,* DnaK*,* GrpE*,* GyrB*,* GroEL*,* YchF*, and *SecY* genes (Devulder et al. 2005; Guo et al. 2008; Adékambi et al. 2011). Devulder et al. (2005) used a multigene approach to phylogenetic study of mycobacteria species. The phylogenetic relationships of *Frankia* strains were also analyzed using the gene *rpoB*-based classification technique (Bernèche-D’Amours et al., 2011). *Kribbella*-type strains were distinguished using the *gyrB* gene (β-subunit of DNA gyrase), which produced a superior resolution than the *16 S rRNA* gene (Kirby 2011). Actinobacteria and allied taxa’s morphological and physiological traits are depicted in Fig. 5.

For instance, The introduction of several new species into established genera and new genera into the sub-order Micrococcineae has altered the branching order of certain families. As a result, the genera *Rarobacter* and *Sanguibacter* have been removed from the families *Cellulomonadaceae* and *Intrasporangiaceae*, respectively, and *Dermatophilaceae* has been dissected (Stackebrandt and Schumann 2000).

**Fig. 6:**
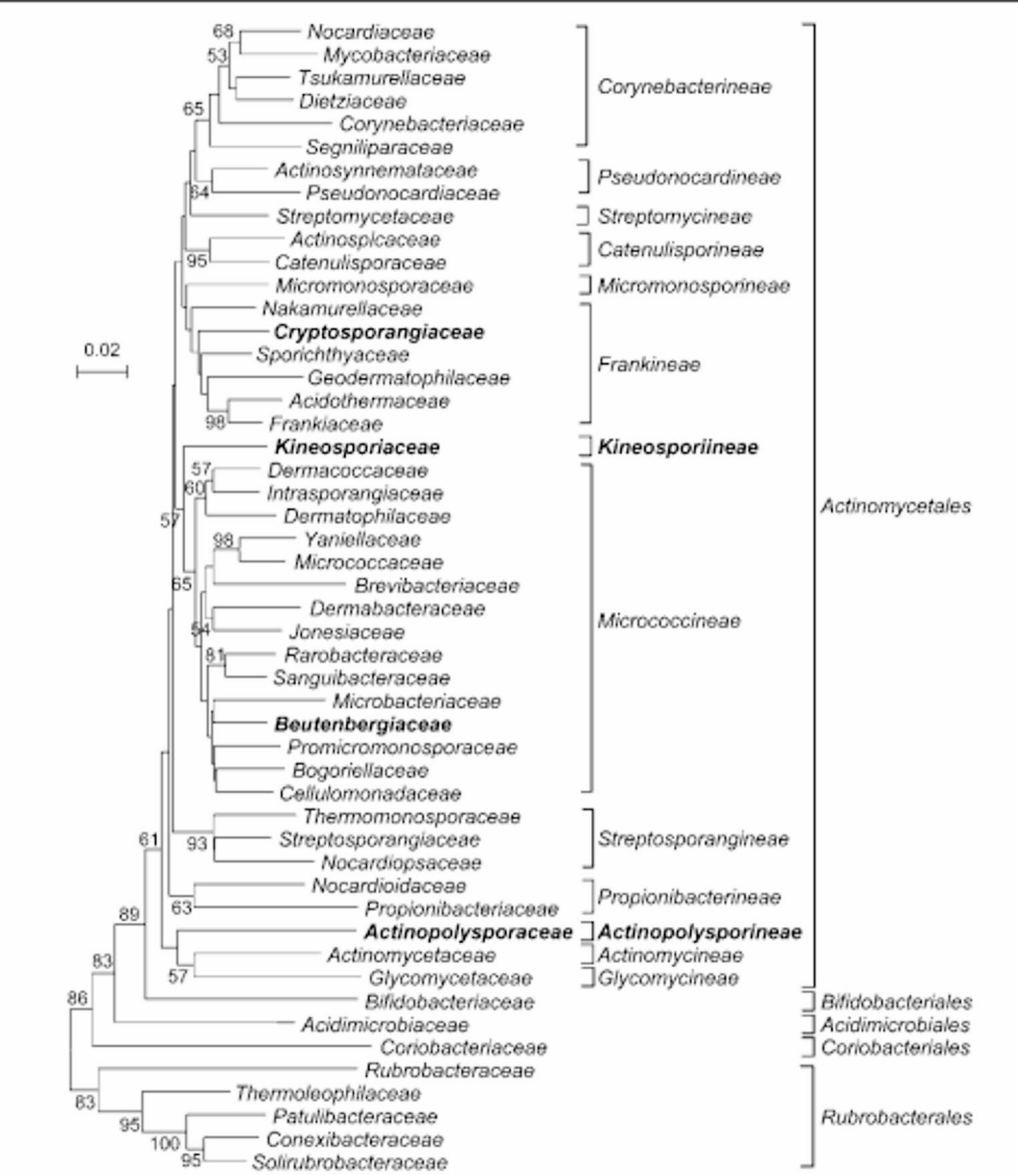
Intraclass relatedness of the class Actinobacteria showing the presence of five orders based on 16 S rRNA gene sequence comparison. The phylogenetic relatedness of the families of the class Actinobacteria is outlined. Taxa newly described in this paper are highlighted in bold. Bootstrap values of 50% or more are indicated at branch points. Bar, 2 substitutions per 100 nucleotide positions. (Xiao et al. 2009)

The continuous but inevitable modifications of the phylogeny-based approach to maintaining categorization in step with discoveries into natural relationships may confuse novices to the field, even though taxonomists know that revisions are an inherent feature of any classification technique Fig. 6.

### Actinobacteria in bioremediation

According to recent studies, bacteria and fungi are essential in breakdown of cellulose and lignocellulose in soil conditions (Baldrian et al., 2012; Berlemont and Martiny 2013). Genes involved in the breakdown of cellulose are most abundant in the genomes of bacteria belonging to the phylum Actinobacteria **(**Berlemont and Martiny 2013**).**

Several isolates have demonstrated the ability to efficiently break down polysaccharides and polyphenols. To decrease the effects of contaminants, a variety of bioremediation agents, including nutritional additions, enzymes, and microbiological cultures, accelerate biodegradation **(**Anderson 2012; Enkhbaatar et al. 2012; Větrovský et al. 2014**).**

Actinobacteria can survive in stressful environments, such as heavy metal-contaminated soils, due to their filamentous mycelial growth, spore-forming ability, and robust secondary metabolism. As a result, these bacteria have evolved several resistance mechanisms **(**Bajkic et al. 2013a).

Actinobacteria are considered highly effective in the breakdown of lignocellulose in environments contaminated with heavy metals, where they may be the primary metabolically active species. Previous studies have shown that while the abundance of other bacterial phyla typically decreases as heavy metal bioavailability rises, the quantity of Actinobacteria remains equal to or even higher than in clean soils with similar features (Gremion et al. 2003; Berg et al. 2012).

In Actinobacteria, heavy metal resistance is strongly influenced by genetic regulatory networks that coordinate the expression of metal-responsive operons. For instance, in *Streptomyces* spp., the ars operon (arsRBCDA) is activated when ArsR, a transcriptional repressor from the ArsR/SmtB family, binds arsenite and releases DNA, thereby allowing transcription of arsenic detoxification genes (Xu et al. 2017). Likewise, mercury detoxification is governed by the mer operon (merRTPCADE), where MerR functions as a dual regulator that senses Hg²⁺ and induces transcription of genes encoding mercuric reductase and transport proteins (Brown et al. 2003). In *Micromonospora* and related taxa, resistance to cadmium, zinc, and cobalt is mediated by the czcCBA efflux pump, which is under the control of the two-component regulatory system *CzcRS* that modulates gene expression in response to metal ion concentration (Nies 2003). Recent genomic analyses further revealed that Actinobacterial genomes harbor diverse metalloregulator gene families (e.g., ArsR, MerR, CsoR, NikR), which integrate metal sensing with transcriptional regulation, ensuring rapid adaptation to fluctuating environments (Santos et al. 2020). Thus, the genetic regulation of these operons provides a molecular basis for Actinobacterial resilience in metal-contaminated habitats.

### Bioremediation strategies by actinobacteria

Actinobacteria utilize a range of mechanisms to mitigate environmental pollution, making them valuable candidates for eco-friendly remediation technologies. One key process is adsorption, wherein pollutants particularly heavy metals are captured on the surface of the microbial cell wall through interactions with reactive functional groups like hydroxyl, carboxyl, and phosphate moieties. This binding mechanism effectively decreases the mobility and toxicity of these contaminants in soil and water systems (Ali et al. 2021). In addition, enzymatic degradation represents a vital strategy through which Actinobacteria secrete diverse extracellular enzymes including hydrolases, laccases, and peroxidases that can break down complex and persistent organic pollutants such as synthetic dyes, PAHs, and pesticides. For instance, species within the *Streptomyces* genus are known for producing oxidative enzymes capable of dismantling lignin-like structures and other xenobiotics into harmless metabolites (Kumar et al. 2020).

Moreover, biotransformation processes carried out by Actinobacteria involve the chemical modification of pollutants via microbial metabolism. This may include reactions such as oxidation, reduction, or dehalogenation, leading to the detoxification or mineralization of hazardous compounds. Notably, genera such as *Nocardia and Rhodococcus* have demonstrated remarkable efficiency in transforming hydrocarbons and industrial solvents into less harmful derivatives (Singh et al. 2019). These multifaceted metabolic capabilities underline the ecological significance of Actinobacteria in restoring contaminated environments and support their integration into sustainable bioremediation frameworks **(**Zhou et al. 2022).

Table 3 summarizes representative *Actinomycete* species involved in bioremediation processes, highlighting their associated contaminants and the underlying mechanisms. The diversity of these mechanisms including adsorption, enzymatic degradation, and transformation demonstrates the metabolic versatility of Actinobacteria in addressing a wide range of environmental pollutants.

**Table 3:** Actinomycetes and bioremediation mechanisms

Actinomycete species	Contaminant	Mechanism
*Streptomyces griseus*	Heavy metals	Adsorption (binding to metal ions)
*Streptomyces albus*	Pesticides	Enzymatic breakdown (hydrolysis)
*Nocardia asteroides*	Hydrocarbons	Transformation (biodegradation)
*Rhodococcus sp.*	Phenolic compound	Enzymatic breakdown (oxidation)
*Actinomyces israelii*	Organic solvents	Adsorption (physical binding)

Recent advances indicate that combining plants with targeted microbial inoculants (bioaugmentation-assisted phytoremediation) substantially improves heavy-metal removal from contaminated soils, by enhancing metal uptake, stabilization, and plant tolerance. Kurniawan et al. (2022) reviewed practical limitations and the promising role of bioaugmentation and plant growth-promoting bacteria in phytoremediation, highlighting the potential for microbe-assisted strategies to overcome field-scale constraints. Actinobacteria in particular have emerged as effective plant-associated partners: they tolerate metal stress, produce siderophores and metal-binding metabolites, and express enzymes (e.g., ACC deaminase) that alleviate metal-induced phytotoxicity and promote root growth. These traits make genera such as *Streptomyces* and *Micromonospora* attractive candidates for enhancing phytoremediation performance. Integrating these microbial mechanisms with phytoremediation could therefore strengthen remediation outcomes and field applicability.

### Environmental bioremediation of metal and organic contamination

Heavy metal contaminants can accumulate in biological systems, leading to toxicity by changing the structural arrangement of proteins and nucleic acids and interfering with oxidative phosphorylation. At higher concentrations, these metals can have severe ecological consequences and pose significant risks to human and animal health **(**Meena et al. 2005; Yaoa et al. 2008).

Certain heavy metals, such as chromium (Cr), lead (Pb), cadmium (Cd), and mercury (Hg), are known for their carcinogenic, mutagenic, and cytotoxic properties. Their toxic effects on the kidneys and nervous system can manifest as neurological disorders, fatigue, headaches, abdominal cramps, diarrhea, and anemia. In severe cases, prolonged exposure can lead to irreversible damage to cellular organelles. Additionally, although essential trace elements at low concentrations, metals such as copper (Cu), nickel (Ni), cobalt (Co), and zinc (Zn) can reach hazardous levels due to industrial activities, posing significant health risks globally **(**Wang et al. 2009).

Among the most concerning organic pollutants are pesticides, including dichlorodiphenyltrichloroethane (DDT), hexachlorobenzene (HCB), and hexachlorocyclohexane (HCH) isomers. Studies have shown that HCB levels are significantly higher in urban areas than in remote locations, suggesting that anthropogenic activities contribute to the widespread contamination by HCHs and DDTs **(**Torre et al. 2016).

**Fig. 7:**
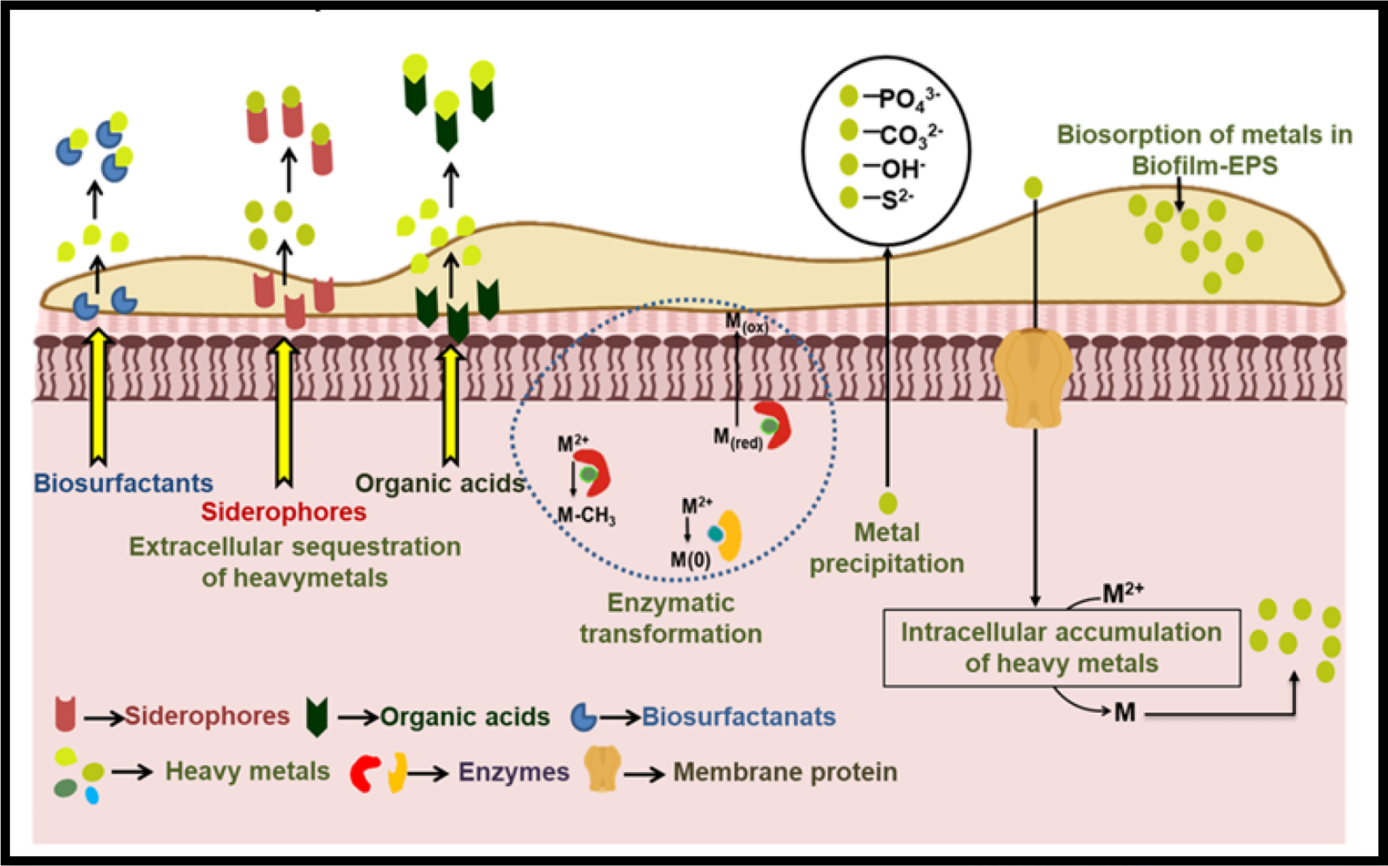
Actinobacteria have adapted several key techniques for heavy metal bioremediation. Biosurfactants, siderophores, and organic acids are the primary means of mobilising heavy metals for bioremediation, while biosorption, bioprecipitation, and bioaccumulation are the methods used to immobilise heavy metals. **(** Shivananda and Surajit 2023**)**

Actinobacteria, particularly *Streptomyces* spp., have demonstrated a strong potential for heavy metal bioremediation from wastewater and explained further in Fig. 7. According to Majdah and Ahmed (2016**)**, *Streptomyces* spp. were capable of removing various heavy metals, with reported removal efficiencies of Cd²⁺ (12%), Cr²⁺ (22%), Cu²⁺ (16%), Fe²⁺ (24%), Ni²⁺ (12%), Zn²⁺ (11%), Mn²⁺ (79%), and Pb²⁺ (32%).

### Mechanisms of bioremediation

**Fig. 8:**
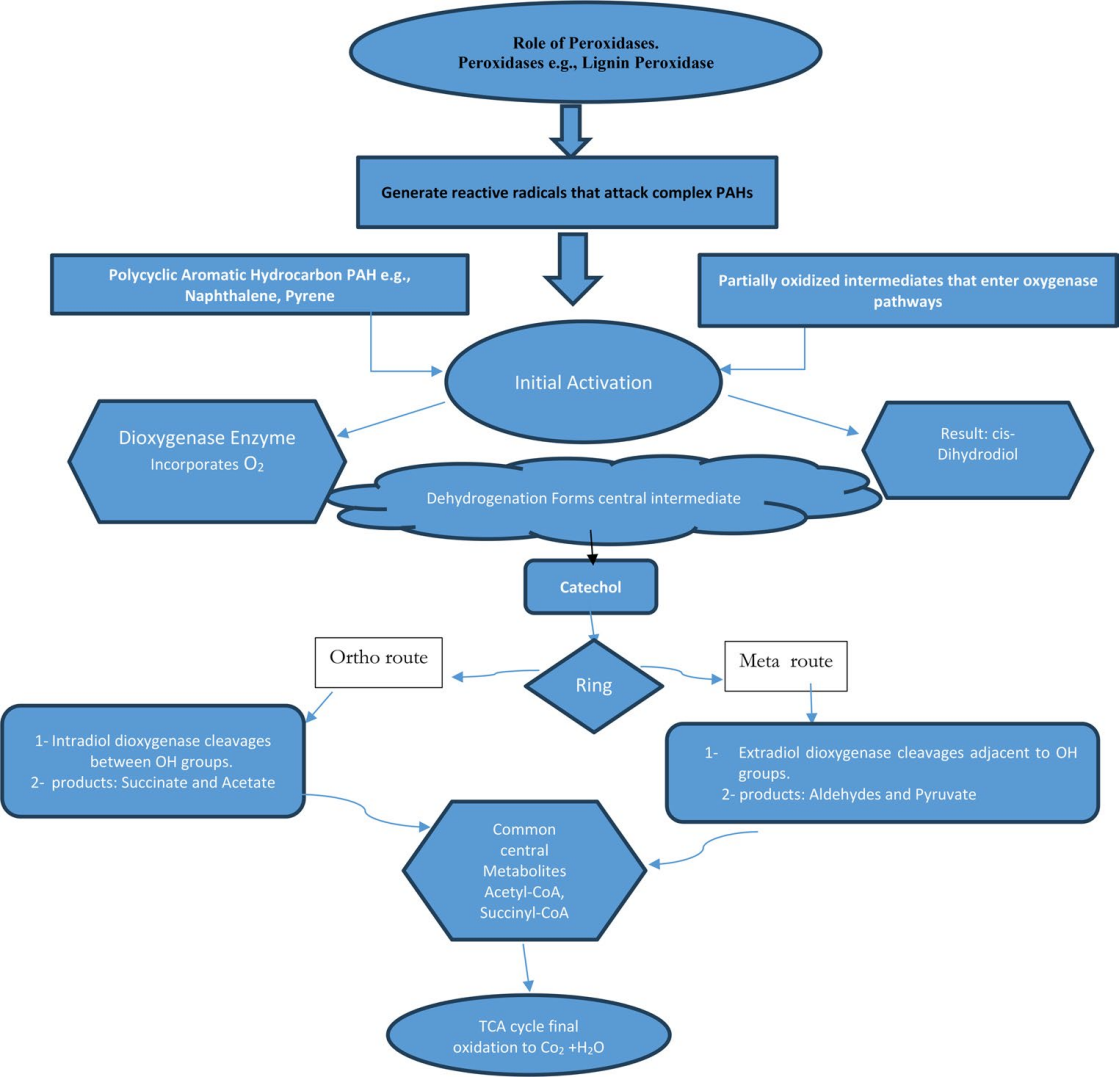
Schematic diagram of PHA degradation pathway showing the sequential action of key enzymes

**Fig. 9:**
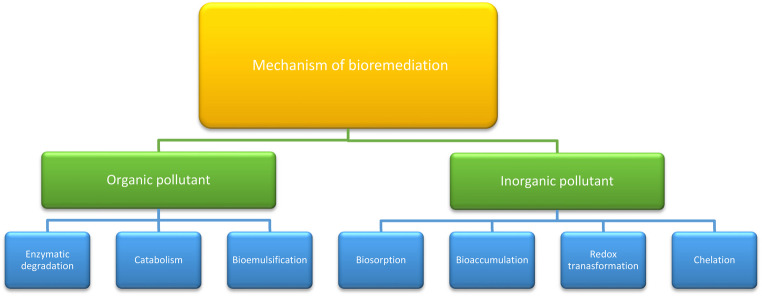
Mechanisms of bioremediation by Actinomycete

Microbial processes facilitate heavy metal bioremediation by altering their oxidation states. This occurs through two primary mechanisms: production of degradative enzymes that target specific pollutants, and development of resistance mechanisms against heavy metals (Berlemont and Martiny 2013; Bajkic et al. 2013b). In addition to these general mechanisms, Actinobacteria exhibit a wide range of synergistic bioremediation strategies, making them particularly efficient in detoxifying contaminated environments. These include: Regarding enzymatic degradation of organic pollutants, Actinobacteria produce extracellular oxygenases, hydrolases, and peroxidases that break down complex compounds, such as polycyclic aromatic hydrocarbons (PAHs), into less harmful intermediates. The schematic diagram showing in Fig. 8 illustrates the proposed pathway of PAH degradation by Actinobacteria, in which oxygenases (such as dioxygenases and monooxygenases) catalyze the initial hydroxylation of aromatic rings, producing cis-dihydrodiol intermediates that are further converted into catechol derivatives. These intermediates undergo subsequent ring-cleavage reactions, mediated mainly by peroxidases, yielding muconic acid and other metabolites that are funneled into central metabolic pathways, including the tricarboxylic acid (TCA) cycle, for complete mineralization. Regarding metal biosorption and bioaccumulation, secreted siderophores and cell wall structures, which are abundant in peptidoglycan and extracellular polymeric substances (EPS), aid in the chelation and immobilization of harmful metals. Genetic regulation, The presence of catabolic gene clusters and regulatory networks allows Actinobacteria to sense pollutants and efficiently upregulate relevant degradative enzymes.These combined mechanisms reinforce the ecological versatility and remediation potential of Actinobacteria in both organic and inorganic pollutant contexts.The following Fig. 9 outlines the mechanisms by which Actinobacteria contribute to the bioremediation of both organic and inorganic pollutants.

### Current advances and innovative strategies

Recent technological progress has significantly expanded both the understanding and application of Actinobacteria in environmental bioremediation. Key advancements include:

- Omics-Driven insights
  High-throughput approaches such as metagenomics, transcriptomics, and proteomics have unveiled novel degradative pathways and intricate regulatory networks in Actinobacteria. These multi-omics tools facilitate the discovery of catabolic gene clusters involved in the breakdown of persistent pollutants and enable the rational engineering of strains with enhanced degradation capabilities.
- Emerging technological strategies
  - *Immobilized-cell systems and nanomaterials*: Encapsulation of Actinobacteria in biocompatible matrices or conjugation with nanoparticles enhances cell stability and promotes effective pollutant interaction, thus improving removal efficiency.
  - *Biosurfactant production*: Biosurfactants derived from Actinobacteria enhance the bioavailability of hydrophobic contaminants, thereby accelerating enzymatic degradation and facilitating access to otherwise inaccessible pollutants.

Bioremediation can proceed via two distinct pathways:

- **Direct reduction**
  Metal reductase enzymes are used in the ex-situ process of direct reduction. But in general, this approach to metal extraction is ineffective. It can be used for soil remediation after excavation (such as pulping, piling, and inoculation with a suitable microbial consortia), but it is mostly useful for groundwater decontamination using bioreactors. **(**Macaskie et al. 2005)
- **Indirect reduction**
  It is an in-situ approach that leverages sulfate-reducing bacteria (*SRB*) to facilitate metal precipitation. This method is considered environmentally sustainable and cost-effective, serving as an alternative to traditional pump-and-treat methods for contamination of groundwater and excavation-based soil treatment. In this process, substrates are injected into subsurface zones to promote microbial growth, leading to the biological generation of *H₂S*, which reacts with dissolved metals, thereby immobilizing them. **(**Macy et al. 1993)

### Role of actinobacteria in metal bioremediation

#### Biosorption and bioaccumulation

The process by which negatively charged microbial cell membranes bond to positively charged heavy metal ions (cations) is known as biosorption. Metal sequestration is facilitated by the capsule-forming properties of bacterial polysaccharides released as extracellular slime. Following surface adsorption, metal ions are transported into the cytoplasm via transporter proteins, leading to intracellular bioaccumulation. **(**Volesky 2007**)**

#### Immobilisation catalysed by biology

Within microbial cells, metal ions undergo enzymatic reduction and become immobilized as iron (Fe)-oxides or organic colloids. This process prevents their mobility and potential toxicity in the environment. **(**Schmidt et al. 2005)

#### Metal detoxification via valence transformation

**Fig. 10:**
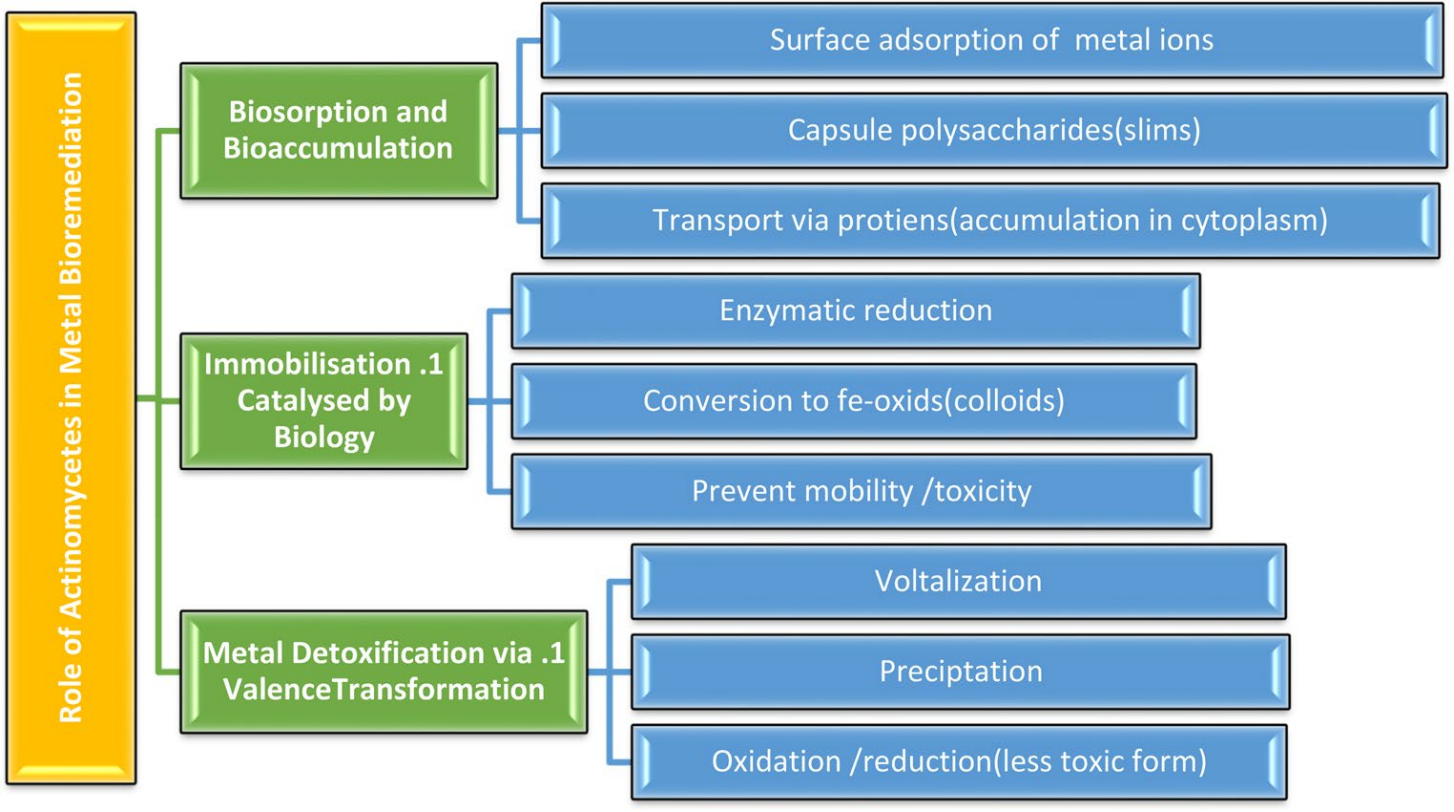
Role of Actinomycetes in heavy metal bioremediation

Actinobacteria can detoxify heavy metals through volatilization, extracellular chemical precipitation, or valence transformation Fig. 10. By altering the oxidation states of metals, actinomycetes can transform toxic metal ions into less hazardous or immobilized forms. For instance, several *Streptomyces* species have been shown to detoxify cadmium and lead through extracellular precipitation, forming stable metal complexes. Likewise, *Arthrobacter* spp. are capable of reducing hexavalent chromium [Cr(VI)] to the less toxic trivalent form [Cr(III)], highlighting their role in valence transformation. In addition, *Nocardia* spp. and related genera can volatilize selenium and mercury, thereby decreasing their bioavailability and toxicity in contaminated environments. **(**Macaskie et al. 2005).

#### Bioremediation of petroleum refinery effluent

Petroleum consists of a complicated blend of hydrocarbons, such as benzene, toluene, naphthalene, and benzopyrene, along with various resins. The majority of these compounds exhibit stability and possess toxic and cancer-causing properties. **(**Yemashova et al. 2007). Proteobacteria such as *Pseudomonas* species and *Azotobacter vinelandii* effectively broke down petroleum hydrocarbons in a consortium, achieving degradation values between 66.83 and 69.6%. Hydrocarbons transform into carbon dioxide and water while releasing energy and cellular mass, which supports the growth and functions of microbes **(**Onwurah and Nwuke 2004**).** *Pseudomonas* species broke down diesel fuel by eliminating long and medium-chain alkanes **(**Ghazali et al. 2004). *Actinomadura* species, *Brevibacillus* species, and an unidentified bacterial clone formed a microbial group that improved oil extraction and biopolymer generation through various treatment methods established by Jing et al. (2008).

#### Bioremediation through biosurfactants and biosorption

Biosurfactants can be found naturally in the ground, demonstrating superior compatibility with living organisms and effective breakdown by microbes **(**Calvo et al. 2009). Various microorganisms produce an extensive array of surface-active agents (SAAs), which are categorized based on their molecular sizes, characteristics, and locations (Pacwa-Plociniczak et al. 2011). The influence of microbial surfactants with both low and high molecular weights was documented by (Banat et al. 2010). Biosurfactants consist of small molecular compounds (SACs) capable of reducing the surface tension between air and water interfaces, exhibiting surface activity and consequently enhancing the surface area of organic substances that insoluble **(**Abdel-Mawgoud et al. 2010). Bioemulsifiers consist of large molecular structures which excel in maintaining the stability of oil-in-water emulsions. This enhancement leads to a greater surface area accessible for the process of bacterial biodegradation.

The removal or breakdown of organic contaminants through bioremediation or biodegradation is enhanced by introducing bioemulsifiers and biosurfactants into the ecosystem **(**Andrea et al. 2010). The interaction of microbial cells with immiscible hydrocarbons in SASs is influenced by several mechanisms: (i) emulsification, (ii) micellarization, (iii) the attachment and detachment of microorganisms to hydrocarbons, and (iv) the release of pollutants. By using surfactants, the ability of petroleum substances to dissolve is enhanced, as biosurfactants from diesel fuel boost oil motion and can improve bioavailability, thus accelerating biodegradation processes **(**Bordoloi and Konwar 2009**).** Surfactants are defined by their critical micelle concentration (CMC), hydrophilic-lipophilic balance (HLB), and their chemical composition **(**Van Hamme et al. 2006). The critical micelle concentration is influenced by the structure of surfactants, the composition of conductivity or turbidity, temperature, ionic strength, and the types and presence of organic substances within the solutions **(**Fuguet et al. 2005). Micelles have the ability to encapsulate water-repelling impurities within their nonpolar center, leading to a greater seeming solubility of these pollutants in water. **(**Prak and Pritchard 2002**).**

*Nocardiopsis* B4 Actinobacteria has been identified as a possible strain for biosurfactant production by utilizing olive oil as a carbon source and phenyl alanine as a nitrogen source. The highest yield of the biosurfactant from *Nocardiopsis* was achieved with a carbon to nitrogen ratio of 2:1, and the optimal conditions for bioprocessing were found to be a pH of 7.0, a temperature of 30 °C, and a salt concentration of 3%. This specific strain is essential in breaking down PAH compounds in contaminated soils. The biosurfactant’s activity maintained stability over a broad range of temperatures, pH levels, and salt concentrations, thereby highlighting its potential effectiveness for bioremediation purposes.**(**Khopade et al. 2012).

Actinobacteria play a significant role in environmental remediation due to their versatile metabolic capabilities, including the degradation of complex and recalcitrant pollutants such as hydrocarbons, pesticides, dyes, and heavy metals **(**Sarma et al. 2020; Jog et al. 2021). Their ability to produce a wide array of extracellular enzymes (e.g., laccases, peroxidases, monooxygenases) and biosurfactants enhances their effectiveness in bioremediation processes, especially in soil and aquatic environments. Species from genera such as *Streptomyces*, *Rhodococcus*, and *Nocardia* have shown notable potential in degrading petroleum compounds, synthetic dyes, and chlorinated hydrocarbons under various environmental conditions **(**Elazzazy et al. 2015). While bacteria (e.g. *Pseudomonas*) and fungi (e.g. *Trichoderma*) are extensively used in bioremediation, Actinobacteria offer unique capabilities in extreme environments but remain underexplored in field-scale applications.

However, most existing studies remain limited to lab-scale experiments using pure cultures under controlled conditions. These findings often fail to translate into real-world applications due to environmental complexity, microbial competition, and fluctuations in physical-chemical parameters **(**Sarma et al. 2020). Additionally, challenges such as slow growth rates, sensitivity to extreme pH or salinity, and difficulty in maintaining high cell viability in field conditions hinder their practical application.

Importantly, recent technological advances such as genetic engineering, CRISPR/Cas9-mediated genome editing, and synthetic biology remain underutilized in the context of enhancing the bioremediation potential of Actinobacteria. Only a handful of studies have explored the manipulation of biosynthetic gene clusters or stress response pathways to improve degradation efficiency or resistance to pollutants **(**Alvarez et al. 2022). Furthermore, omics-based approaches—metagenomics, transcriptomics, and proteomics—offer powerful tools to study functional gene expression in situ and optimize environmental performance, yet they are rarely integrated into bioremediation strategies involving Actinobacteria **(**Tanaka et al. 2018).

To bridge this gap, future research should focus on:

- Developing multi strain consortia that combine Actinobacteria with other microbes to enhance synergistic degradation.
- Applying bioinformatics and systems biology tools to model and optimize metabolic networks involved in pollutant breakdown.
- Engineering stress tolerant strains using advanced molecular tools for use in harsh and contaminated environments.

By addressing these gaps, Actinobacteria can move beyond laboratory curiosities into practical agents of sustainable environmental remediation.

#### Phytoremediation

Phytoremediation represents a promising and eco-conscious technique for detoxifying environments contaminated with hazardous substances, particularly soils impacted by heavy metals and organic pollutants. Its effectiveness is not solely dependent on plant species, but also significantly influenced by the complex interactions between plant roots and soil microbial communities. Among these beneficial microbes, Actinobacteria have emerged as important allies. These filamentous bacteria enhance phytoremediation efficiency by improving plant resilience to environmental stressors and optimizing nutrient assimilation in challenging conditions. Through the secretion of growth-promoting compounds like phytohormones and siderophores, Actinobacteria not only stimulate root development but also facilitate the release and uptake of otherwise immobilized contaminants. Furthermore, they contribute to the breakdown of organic pollutants and assist in the detoxification or conversion of heavy metals into less biohazardous forms, thus increasing their availability for plant absorption. This symbiotic relationship underscores the potential of Actinobacteria as microbial enhancers in sustainable remediation frameworks. Looking forward, integrating Actinobacteria into phytoremediation practices may revolutionize eco-friendly strategies for soil restoration and agricultural sustainability.

Phytoremediation represents an economical method that employs vegetation to eliminate, change, or neutralize pollutants. In recent years, this approach has been moving towards extensive modeling through a range of mathematical frameworks. These models can serve as valuable resources for enhancing our comprehension and forecasting the factors that affect the efficacy of phytoremediation, allowing for accurate planning of large-scale initiatives. **(**Jaskulak et al. 2020).

Phytoremediation has garnered significant interest recently due to its economical nature, eco-friendly characteristics, and simplicity in addressing soils contaminated with heavy metals. However, there are certain limitations associated with the use of phytoremediation techniques **(**Silambarasan et al. 2022). Phytoremediation involves cultivating vegetation in contaminated settings throughout the entire growth cycle. The roots of these plants take in toxins from the ground and hold them within the rhizosphere, making them safe by stopping their movement into the surrounding areas **(**Nguyen and Phan 2023**).**

Phytoextraction plants take in significant amounts of metals by generating biomass. They can gather substantial levels of heavy metals through their roots and move them upwards to their above-ground parts. Various elements contribute to this mechanism, including: the availability of metals in the rhizosphere, the process of metal absorption, the retention of metals in the roots, the transfer of metals through the xylem to the shoots, and the plants’ tolerance to metals **(**Chaturvedi and Khurana 2019**).**

Phytoremediation of cadmium facilitated by sunflower plants through *S.tendae* and the enhancement of zinc and cadmium mobility via secondary metal-binding compounds from Salix caprea distinctly illustrate the potential role of endophytic Actinobacteria in the extraction of heavy metals from contaminated soil, while also promoting the movement of metals in soils tainted with heavy metals**(**Zhou et al.  2022).

**Fig. 11:**
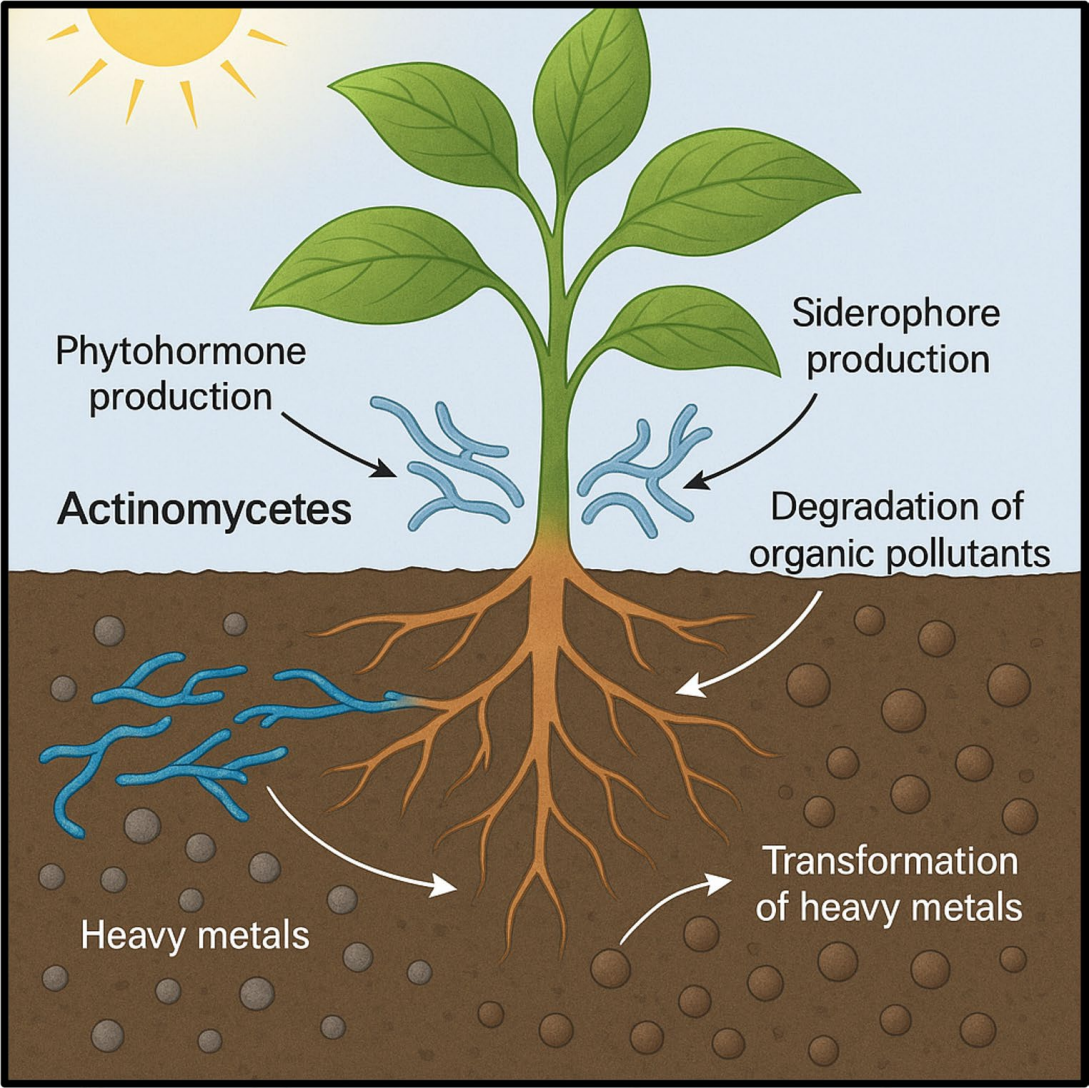
The Role of Actinomycetes in enhancing phytoremediation of contaminated soils

Figure 11 is a conceptual illustration depicting the synergistic role of Actinobacteria in phytoremediation of contaminated soils. Plant roots interact with Actinobacteria in the rhizosphere, where these filamentous bacteria enhance phytoremediation efficiency through multiple mechanisms: (1) secretion of phytohormones and siderophores that stimulate root growth and metal solubilization, (2) transformation of heavy metals into less toxic forms, (3) degradation of organic pollutants, and (4) enhancement of plant stress tolerance and nutrient uptake. This microbial plant cooperation increases contaminant bioavailability and promotes sustainable soil restoration.

#### Factors affecting actinobacteria growth and activity

The growth and bioactive metabolite production of Actinobacteria are shaped by environmental parameters, nutrient sources, and mutagenic treatments.

#### Environmental conditions (pH and temperature)

Actinobacteria generally showed optimal growth and metabolite yield at slightly acidic to neutral pH values (6–7). In starch–casein broth, the highest growth and alkaloid production (240 µg/mL) were observed at pH 6 (Pudi et al. 2016). Strain-specific experiments on starch–nitrate agar indicated that AC12 and AC9 grew best at pH 6.5 while AC2 preferred pH 7.0 (Muleta and Assefa 2018). Temperature tolerance varied among strains: moderate temperatures (30 °C) supported good growth and alkaloid production in some isolates (Pudi et al. 2016), whereas other work reported maximal antibacterial/antifungal activity at 35–40 °C (AC2 at 35 °C; AC9 and AC12 at 40 °C) (Muleta and Assefa 2018).

#### Nutrient sources (carbon and nitrogen)

Carbon source composition strongly influenced alkaloid and antimicrobial yields. Media containing starch and casein supplemented with dextrose produced the highest alkaloid levels (up to 270 µg/mL) compared with other carbon sources (Pudi et al. 2016). Some isolates demonstrated broad carbon-utilization and salinity tolerance (e.g., TG01) (Sudiana et al. 2020), while other work reported elevated antibacterial/antifungal metabolite production in starch-rich media followed by glycerol, D-xylose, D-glucose, D-fructose, sucrose, and maltose (LCHAACC13) (Chali et al. 2021). Regarding nitrogen, inorganic salts (KNO₃, NaNO₃, (NH₄)₂SO₄, NH₄NO₃) generally enhanced antimicrobial activity more than organic sources (urea, casein, yeast extract, peptone); KNO₃ and NaNO₃ were among the most effective. (Al-Zahrain, 2007; Refaat et al. 2017)

#### Salt tolerance

Actinomycete isolates grew at NaCl concentrations up to 5%, with 2% NaCl being optimal for growth and antibiotic production; growth ceased at 7–10% NaCl. (Niemhom and Thawai 2020; Rakesh et al. 2013)

The ability of Actinobacteria to survive under extreme environmental conditions such as acidic pH, high salinity, or nutrient limitation not only enhances their ecological fitness but also often triggers the synthesis of stress related secondary metabolites. These compounds, including biosurfactants, chelators, and enzymes, play a vital role in pollutant solubilization and degradation, making Actinobacteria more effective in bioremediation compared to other microbial groups. Unlike many conventional bacteria, Actinobacteria can adapt to suboptimal conditions, and such stress environments can act as cues for the upregulation of genes involved in biodegradation and detoxification pathways.

Based on previous studies, it becomes clear that Actinobacteria exhibit significant ecological and metabolic diversity that directly influences their bioremediation capacities. The following Table 4 presents a comparative overview linking the environmental origin of Actinomycete strains to specific pollutants and their respective degradation mechanisms. This structured synthesis highlights how the source environment shapes microbial adaptation and function.

**Table 4:** Comparative analysis of actinomycetes from diverse environments

Environmental source	Genus/species	Pollutant type	Bioremediation mechanism	Remarks
Heavy metal-contaminated soil	*Streptomyces griseus*	Lead (Pb), Cadmium (Cd)	Biosorption, production of resistance proteins	High metal resistance; enzymatic detoxification observed
Marine sediments	*Salinispora tropica*	Halogenated organic compounds	Enzymatic degradation (halogenases)	Unique adaptation to marine pollutants
Pesticide-contaminated agricultural soil	*Nocardia sp.*	Atrazine (herbicide)	Enzymatic degradation (hydrolases)	Effective pesticide degradation in soil systems
Desert soil	*Actinomadura sp.*	Petroleum hydrocarbons	Bioemulsification, aerobic oxidation	Resilient in nutrient-poor, arid environments
Industrial dye-contaminated soil	*Micromonospora sp.*	Azo dyes	Reductive cleavage via azoreductases	High activity under low-oxygen conditions

#### Challenges in using actinobacteria for bioremediation

The employment of Actinobacteria in bioremediation has many constraints that may restrict its efficiency in particular settings. One major limitation is their slow growth rate compared to bacteria, which reduces their effectiveness in time-sensitive remediation processes. Furthermore, environmental factors like pH, temperature, and moisture levels have a significant impact on their activity and may not always be ideal in contaminated sites, which limits their performance in natural settings **(**Mohan and Modestra 2021**).**

A further challenge is the competition with native microbial communities in contaminated environments, which can limit the dominance and effectiveness of Actinobacteria in biodegradation processes; additionally, some strains may lack the essential enzymes required to degrade specific pollutants, highlighting the need for genetic modifications or metabolic optimisation to enhance their bioremediation potential; and finally, despite promising laboratory studies, there are still no large-scale field applications, requiring additional validation and real-world testing to confirm their efficacy in soil and water remediation.

According to recent research, Actinobacteria may play a much larger role in bioremediation if growth conditions are improved and cutting-edge methods like genetic engineering and metabolic pathway optimisation are incorporated. This would open the door for a wider use of Actinobacteria in the removal of both organic and inorganic contaminants (Taj and Rajkumar, 2016; Silambarasan et al. 2022).

According to recent studies, Actinobacteria present extra difficulties, such as limitations on genetic modification, which make even simple genetic engineering methods more difficult and time-consuming than for other microorganisms. However, new chances to improve their bioremediation potential are presented by developments in CRISPR/Cas9-based genome editing and whole-genome sequencing employing long-read technologies like PacBio and Oxford Nanopore **(**Gupta et al. 2023).

Furthermore, because of their slow growth rate and intricate nutritional needs, isolation and cultivation issues continue to be major hurdles. To improve their recovery from environmental samples, specialised medium and pre-treatment techniques are required **(**Kim et al. 2022).

While Actinobacteria have demonstrated substantial bioremediation potential in laboratory studies, several critical challenges hinder their effective application in real-world environments. A key limitation lies in their relatively slow growth rate and filamentous morphology, which reduce their competitiveness against fast-growing native microbes in contaminated sites. Moreover, Actinobacteria often exhibit reduced tolerance to environmental stressors such as fluctuations in pH, salinity, and the presence of heavy metals, all of which can compromise their metabolic activity and survival in situ.

The shift from controlled laboratory conditions to complex environmental matrices also raises concerns regarding their ecological fitness and long-term functionality. Many strains fail to adapt or maintain stable populations once introduced into heterogeneous field conditions, especially without adequate carrier systems or nutrient support. Additionally, the genetic regulation of stress responses and pollutant degradation pathways in Actinobacteria remains poorly characterized, limiting opportunities for targeted genetic enhancement or optimization.

Another practical challenge lies in scaling up their use for field applications. Effective delivery systems such as encapsulation techniques or suitable carrier materials are still underdeveloped for filamentous bacteria like Actinobacteria. Furthermore, the absence of standardized field assessment protocols makes it difficult to evaluate their performance objectively or compare outcomes across different studies and environments. Addressing these obstacles requires not only deeper biological insights but also the integration of materials science, formulation technologies, and field-level engineering solutions to improve their efficacy and reliability in environmental biotechnologies.

Actinobacteria isolated from heavy metal contaminated soils often exhibit enhanced resistance to toxic metals such as cadmium and lead, potentially due to selective evolutionary pressure. Conversely, marine-derived strains tend to possess unique enzymatic pathways enabling them to degrade halogenated organic compounds, reflecting adaptation to marine pollutants.

We propose a conceptual framework linking environmental origin with genotypic adaptation and functional bioremediation capacity in Actinobacteria. According to this model, extreme or contaminated environments drive the selection of metabolic pathways that enable pollutant degradation, suggesting a predictable relationship between source habitat and bioremediation potential.

**Fig. 12:**
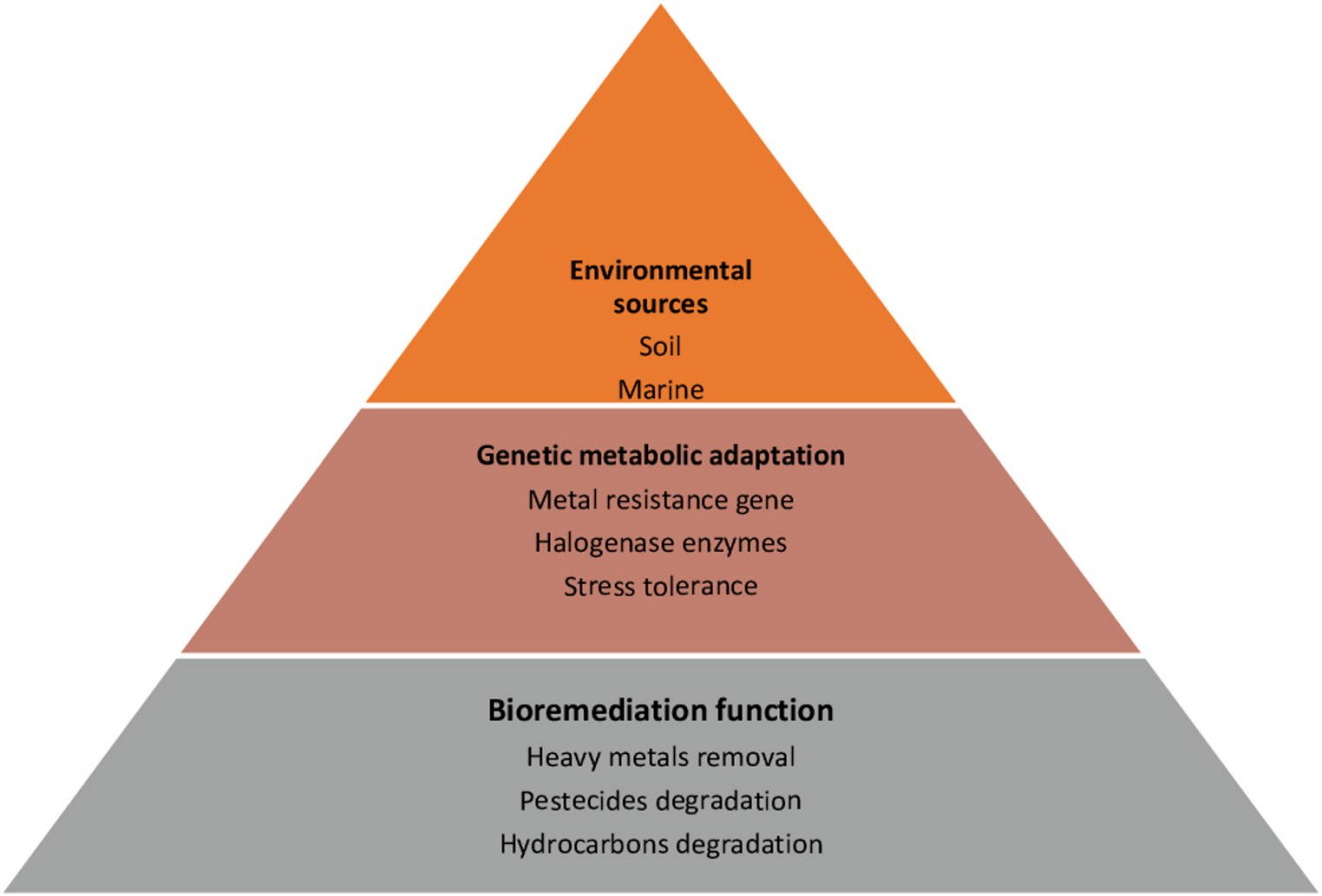
Conceptual model illustrating the linkage between the environmental origin of Actinomycetes, their genetic/metabolic adaptations, and bioremediation functions

Figure 12 illustrates how different environmental niches, including desert soils, marine sediments, and contaminated terrestrial locations, influence the evolution of particular genetic or metabolic features in Actinobacteria. Their ability to break down or change environmental contaminants including heavy metals, pesticides, and hydrocarbons is directly impacted by these adaptations, which include metal resistance genes, halogenase enzymes, and stress tolerance pathways.

Despite the promising laboratory results, the field application of Actinobacteria often encounters significant limitations. For instance, in a study by Zhang et al. (2019), *Streptomyces lydicus* showed a strong antifungal activity against *Fusarium oxysporum* under controlled greenhouse conditions, but failed to suppress the pathogen effectively in open-field trials due to fluctuating environmental parameters and microbial competition.These discrepancies highlight the challenges related to the survivability, colonization efficiency, and metabolic activity of Actinobacteria in complex and variable field environments.

Actinobacteria are metabolically versatile and have demonstrated strong potential in the degradation of persistent pollutants, yet several barriers restrict their translation from laboratory settings to real-world applications. For example, *Streptomyces heliomycini* C1 was reported to degrade up to 83% of the herbicide metribuzin under optimal laboratory conditions (pH 7.2, 30 °C), but such efficiency is rarely replicated under fluctuating field environments (Rebai et al. 2024). Field-scale experiments further indicate that combining bioaugmentation with complementary strategies such as phytoremediation and vermiremediation enhances hydrocarbon removal (up to 76% after 112 days), underscoring the need for consortia-based approaches rather than reliance on single strains (Martínez-Rabelo et al. 2023). However, the survival and sustained activity of Actinobacteria remain vulnerable to abiotic stresses, competition, and resource limitation, challenges that can be mitigated through immobilization on carriers or encapsulation technologies, which have shown improved stability in microbial systems (Ali et al. 2024; Yang et al. 2023). Another limitation arises during scale-up, as degradation efficiency tends to decrease significantly in pilot-scale studies due to poor oxygen diffusion, soil heterogeneity, and limited pollutant bioavailability, highlighting the importance of improved delivery systems and stepwise validation through pilot-scale testing before full deployment.**(** Karthikeyan et al. 2024**)**.

#### Beyond classical genera: rare and extremophilic actinobacteria in bioremediation

In addition to the well-studied genera *Streptomyces*, *Rhodococcus*, and *Arthrobacter*, an increasing body of research has shifted attention toward rare and extremophilic Actinobacteria that possess unique biocatalytic capabilities. Genera such as *Kocuria*, *Nonomuraea*, *Microbacterium*, *Cellulosimicrobium*, *Nocardia*, *Gordonia*, and *Micromonospora* have been reported to degrade dyes, hydrocarbons, plastics, and pharmaceutical pollutants through specialized metabolic pathways not typically encountered in classical taxa (Sharma et al. 2023; Adenan and Ting 2022). Extremotolerant strains from saline, thermophilic, desert, and polar environments further expand this potential, as highlighted by *Nocardiopsis* isolates capable of functioning under high salinity, Antarctic *Streptomyces* with cold-active enzymes, and desert-derived *Microbacterium* strains showing stability under alkaline conditions (Boukhatem et al. 2022; Gorrab et al. 2024).

The enzymatic arsenal of these rare and extremophilic genera has become a focal point for next-generation remediation research. Omics-guided approaches have uncovered novel *laccases*, *peroxidases*, *Rieske oxygenases*, *hydrolases*, and *amidases*, many of which exhibit superior activity and stability under industrially relevant conditions (Makarani and Kaushal 2025; Behera and Das 2023). These findings highlight the importance of expanding the scope of Actinobacterial studies beyond traditional models to incorporate rare and extremophilic taxa, thereby ensuring a more comprehensive and forward-looking framework for bioremediation applications.

#### Future biotechnological applications of actinobacteria

The future of actinomycete research in the context of environmental biotechnology lies in bridging the gap between laboratory findings and field-scale applications. While current studies (Alvarez et al. 2022) have demonstrated their capacity to degrade a range of pollutants and produce valuable metabolites, these findings often remain underexploited due to limitations in scalability, environmental adaptability, and insufficient integration with modern biotechnological tools.

One promising direction is the development of engineered microbial consortia, where Actinobacteria are paired with other microbial partners to improve resilience, degradation efficiency, and nutrient cycling in complex environments. Additionally, the application of machine learning and bioinformatics to predict biodegradation pathways and optimize strain selection could drastically reduce experimental costs and time.(Tanaka et al. 2018).

It is also critical to explore the role of Actinobacteria in multi-stress environments such as polluted sites with fluctuating pH, temperature, and salinity where natural selection may have driven the evolution of unique metabolic traits. These environments could be hotspots for novel gene clusters related to bioremediation or biosynthesis.**(** Tanaka et al. 2018).

On the regulatory and applied side, future studies should aim to establish standardized protocols for field trials, ensuring reproducibility and scalability. Partnerships between academia, industry, and environmental agencies will be key to transforming Actinobacteria from laboratory isolates into reliable tools for environmental clean-up and resource recovery. (Alvarez et al. 2022)

Ultimately, moving from descriptive studies to functional and system-level understanding will unlock the full potential of Actinobacteria in sustainable environmental technologies.

Also, this review proposes a functional classification of Actinobacteria based on their primary bioremediation mechanism: (i) biosorption and bioaccumulation of heavy metals, (ii) enzymatic degradation of organic pollutants, and (iii) redox transformation of toxic compounds. Such a framework provides a clearer understanding of their ecological roles and potential applications. Despite the recognized metabolic versatility of marine-derived Actinobacteria, their role in heavy metal bioremediation remains largely unexplored. Future research should focus on isolating and characterizing strains from deep-sea sediments to uncover novel resistance mechanisms.

Looking ahead, the study of Actinobacteria in the context of bioremediation is poised for exciting developments. Beyond their well-documented metabolic capabilities, emerging perspectives suggest that these microorganisms could play a central role in shaping next-generation environmental recovery strategies.

One promising avenue involves envisioning Actinobacteria as pivotal components of “eco-synthetic systems” integrated frameworks combining microbial biotechnology with smart environmental management. In the face of accelerating climate change and expanding anthropogenic pressures, these systems may incorporate genetically enhanced strains, engineered microbial consortia, and even AI-assisted microbiome designs, with Actinobacteria acting as core bio-agents. Their genomic adaptability, ecological robustness, and enzymatic diversity position them uniquely to move beyond traditional pollutant degradation roles, toward functioning as proactive agents in predictive and programmable environmental remediation.

This forward-looking perspective invites further interdisciplinary research to harness the full potential of Actinobacteria not only as natural degraders but as engineered partners in building resilient, sustainable ecosystems.

Future research should focus on integrating synthetic biology with ecological applications to enhance the utility of Actinobacteria in bioremediation. Advances in genome editing, pathway engineering, and synthetic consortia design can be harnessed to create strains with optimized degradation pathways and improved resilience under variable environmental conditions. A promising direction is the development of Actinobacteria-based systems that not only detoxify pollutants but also recover valuable resources from waste streams. For example, engineered strains could be tailored to simultaneously degrade hazardous organic compounds while mobilizing and concentrating critical elements such as rare earth metals from electronic waste. This dual-function approach has the potential to transform remediation from a purely corrective process into a sustainable strategy that couples environmental restoration with resource recovery. Bridging laboratory innovation with field-scale ecological implementation will require iterative pilot testing, ecological modeling, and close collaboration between microbiologists, engineers, and environmental policymakers.

**Fig. 13:**
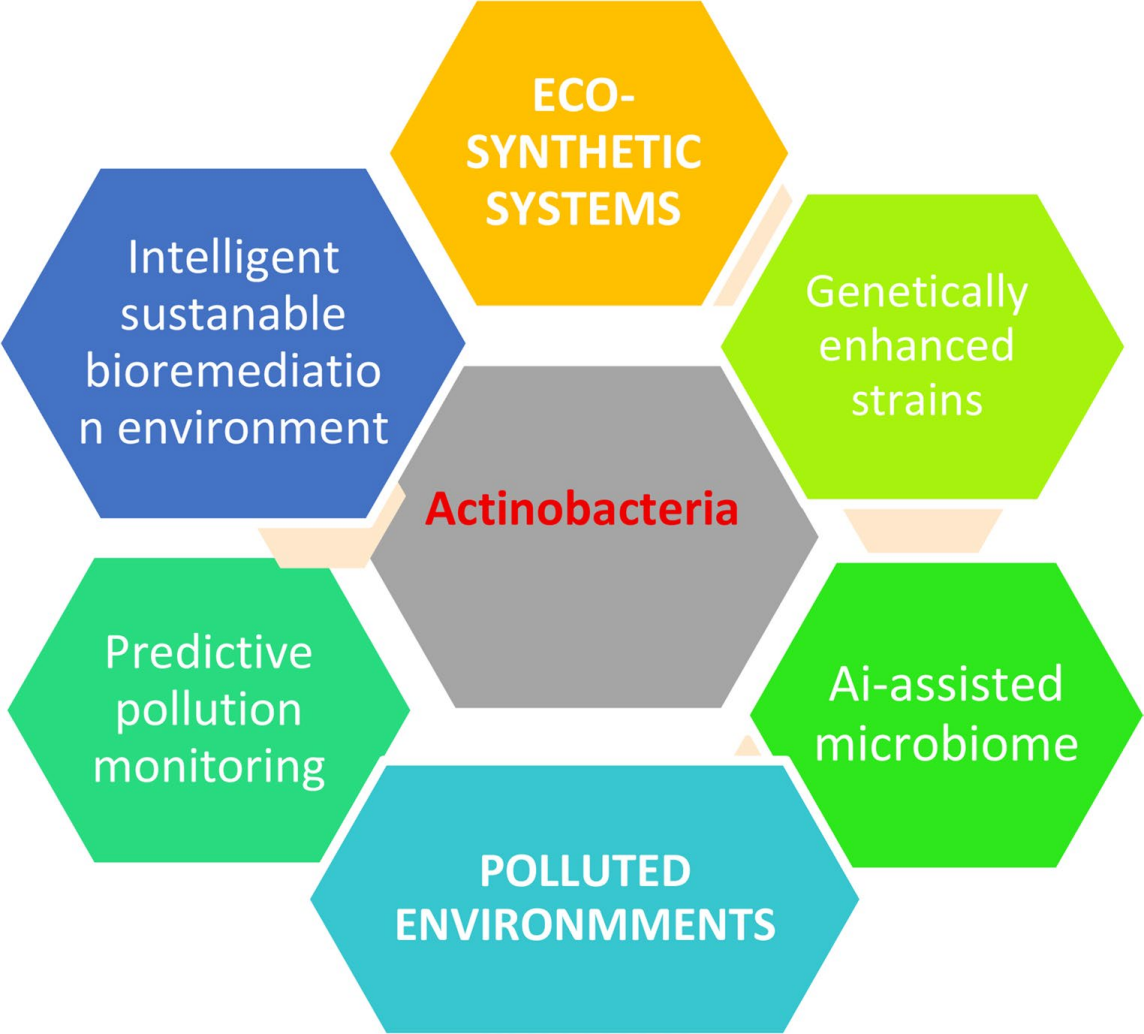
Actinomycetes as eco synthetic system for bioremediation in future

Figure 13 it showing conceptual framework illustrating the envisioned role of Actinobacteria in future eco-synthetic systems for bioremediation. The central circle highlights Actinobacteria as key microbial agents. Supporting elements include genetically enhanced strains, engineered microbial consortia, and AI-assisted microbiome design, all integrated within eco-synthetic systems. These systems are proposed to operate under the pressures of climate change and polluted environments. Expected outcomes include intelligent bioremediation, sustainable environmental recovery, and predictive pollution monitoring. This model emphasizes a forward-looking vision for Actinobacteria as bio-architects in next-generation remediation strategies.

#### Emerging contaminants: pharmaceuticals and personal care products (PPCPs)

Emerging pollutants such as pharmaceuticals and personal care products (PPCPs) have become a growing environmental and public health concern. Recent studies demonstrate that Actinobacteria contribute to the biodegradation of certain PPCPs (Babič et al. 2024). For example, isolates of *Micrococcus luteus*, *Micrococcus yunnanensis*, and *Kocuria rhizophila* from volcanic environments have shown tolerance to—and possibly transformation of—diclofenac, ibuprofen, and paracetamol at low concentrations (Vázquez-Campos et al. 2024). Moreover, selective enrichment experiments from aquatic biofilms revealed that Actinobacterial genera such as *Nocardioides and Pseudonocardia* can effectively degrade ibuprofen and diclofenac, achieving over 90% reduction in concentration under controlled conditions (Xia et al. 2023). Additionally, metagenomic data from membrane bioreactor systems indicate that microbial communities containing Actinobacterial members are capable of degrading caffeine (among other PPCPs), although the dominant degraders may include other genera like *Leucobacter* and *Pseudomonas* (Chen et al. 2023 and Li et al. 2023).

#### Positioning of the present review in relation to recent literature

Several recent reviews (2022–2025) have emphasized the importance of Actinobacteria in bioremediation, yet each adopts a specific perspective. For instance, Adenan and Ting (2022**) **and Behera and Das (2023**)** provided broad overviews of soil-derived strains and their pollutant-degrading pathways, while Fernandes et al. (2024) concentrated on hydrocarbon degradation by marine taxa. Boukhatem et al. (2022) highlighted plant growth-promoting Actinobacteria as agents for stress adaptation, and Gorrab et al. (2024) discussed extremozymes with potential industrial applications. Helmi et al. (2025) examined the diversity and antimicrobial potential of Saudi Arabian isolates, whereas Makarani and Kaushal (2025) summarized recent advances in Actinobacteria-based remediation technologies without offering biodiversity comparisons across ecosystems.

In contrast, the present review uniquely contributes by (i) synthesizing biodiversity patterns of Actinobacteria across terrestrial, aquatic, and extreme ecosystems, (ii) introducing comparative matrices that link taxa with functional attributes and ecological niches, and (iii) proposing explicit translation-to-field criteria. These elements distinguish this work from existing reviews and provide an integrated framework that connects Actinobacterial diversity with applied bioremediation strategies. Also, the present review aims to bridge these gaps by synthesizing biodiversity patterns of Actinobacteria across diverse ecosystems, introducing comparative matrices that connect taxa with their functional attributes and environmental niches, and proposing explicit translation-to-field criteria. In doing so, this work not only consolidates existing knowledge but also provides a practical framework to guide the design and deployment of Actinobacteria-mediated bioremediation strategies.

#### Search strategy and selection criteria

This review followed a narrative approach, drawing on peer-reviewed literature obtained primarily from PubMed, Scopus, Web of Science, and Google Scholar. The keywords used in various combinations included Actinobacteria, actinomycetes, bioremediation, environmental pollution, microbial diversity, heavy metals, hydrocarbons, and synthetic consortia. The emphasis was placed on recent publications between 2020 and 2025, reflecting the rapid expansion of this field and the availability of updated datasets. Nevertheless, earlier seminal studies were also incorporated when they provided unique or historically foundational insights, particularly in relation to taxonomy, biodegradation pathways, or pioneering applications. Only full-text articles, review papers, and original research directly relevant to Actinobacteria and their environmental roles were included, while non-peer-reviewed sources and tangential reports were excluded.

## Conclusion

Actinobacteria continue to represent a cornerstone in microbial biotechnology due to their remarkable ability to synthesize a wide range of bioactive metabolites. Their role in environmental remediation particularly in the detoxification of heavy metals, degradation of hydrocarbons, and production of biosurfactants positions them as sustainable alternatives to conventional remediation technologies. Despite their promise, challenges such as slow growth, limited field-scale validation, and competition with native microbiota remain significant barriers to implementation.

To fully harness their potential, future efforts should focus on optimizing growth conditions, enhancing pollutant degradation pathways through genetic engineering, and developing robust bioprocessing techniques tailored for diverse environmental settings. Emerging tools such as CRISPR-Cas systems, omics-based profiling, and synthetic biology hold great promise for unlocking novel capabilities in Actinobacteria. Collaborative, interdisciplinary research will be essential to transition these organisms from promising laboratory models to effective field-scale bioremediation agents.

## Data Availability

Data will be available upon reasonable request.
